# Corridor-Level Impacts of Battery-Electric Heavy-Duty Trucks and the Effects of Policy in the United States

**DOI:** 10.1021/acs.est.3c05139

**Published:** 2023-12-18

**Authors:** Wilson H. McNeil, Fan Tong, Robert A. Harley, Maximilian Auffhammer, Corinne D. Scown

**Affiliations:** † Energy Technologies Area, Lawrence Berkeley National Laboratory, Berkeley, California 94720, United States; ‡ Department of Civil and Environmental Engineering, University of California, Berkeley, Berkeley, California 94720, United States; § Department of Civil and Natural Resources Engineering, University of Canterbury, Christchurch 8041, New Zealand; ∥ School of Economics and Management, Beihang University, Beijing 100191, People’s Republic of China; ⊥ Lab for Low-carbon Intelligent Governance, Beihang University, Beijing 100191, People’s Republic of China; # Department of Agricultural and Resource Economics, University of California, Berkeley, Berkeley, California 94720, United States; 7 National Bureau of Economic Research, Cambridge, Massachusetts 02138, United States; 8 Life-Cycle, Economics and Agronomy Division, Joint BioEnergy Institute, Emeryville, California 94608, United States; 9 Biosciences Area, Lawrence Berkeley National Laboratory, Berkeley, California 94720, United States; 10 Energy and Biosciences Institute, University of California, Berkeley, Berkeley, California 94720, United States; 11 Peking University Ordos Research Institute of Energy, Ordos City 017000, Inner Mongolia, People’s Republic of China

**Keywords:** Air Pollution, Human Health, Climate Change, Inflation Reduction Act, Freight, Battery-Electric Trucks, Electricity Grid Emissions

## Abstract

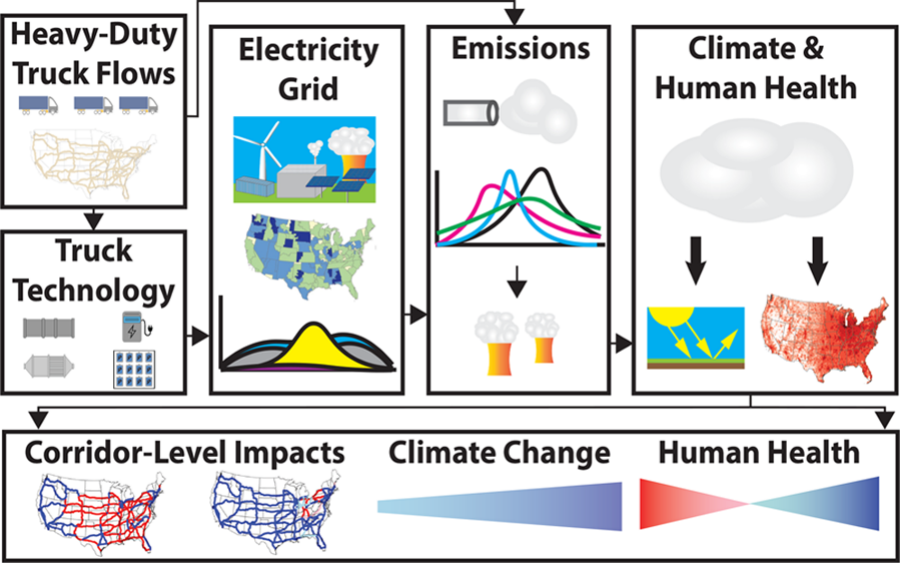

Electrifying freight trucks will be key to alleviating air pollution burdens on disadvantaged communities and mitigating climate change. The United States plans to pursue this aim by adding vehicle charging infrastructure along specific freight corridors. This study explores the coevolution of the electricity grid and freight trucking landscape using an integrated assessment framework to identify when each interstate and drayage corridor becomes advantageous to electrify from a climate and human health standpoint. Nearly all corridors achieve greenhouse gas emission reductions if electrified now. Most can reduce health impacts from air pollution if electrified by 2040 although some corridors in the Midwest, South, and Mid-Atlantic regions remain unfavorable to electrify from a human health standpoint, absent policy support. Recent policy, namely, the Inflation Reduction Act, accelerates this timeline to 2030 for most corridors and results in net human health benefits on all corridors by 2050, suggesting that near-term investments in truck electrification, particularly drayage corridors, can meaningfully reduce climate and health burdens.

## Abstract 2

By employing a corridor-level analysis of heavy-duty truck decarbonization and considering variations in regional energy systems, this study finds that recent U.S. policy (i.e., the Inflation Reduction Act) accelerates grid decarbonization, reducing health and climate impacts from truck electrification.

## Introduction

A reliable and efficient freight transportation system is an essential component of the U.S. economy. Trucking is the cornerstone of global freight movement, transporting far more payload on land than any other mode and providing drayage services that enable rail and maritime shipping.^1,2^ In the U.S., trucks transport 73% of the freight by value and 71% by payload.^1−3^ However, despite accounting for only 10% of total vehicle miles traveled (VMT), medium- and heavy-duty trucks consume 19% of U.S. transportation energy use and emit 25% of on-road carbon dioxide (CO_2_), 55% of fine primary particulate matter (PM_2.5_), and 43% of nitrogen oxides (NO_*x*_).^2^ Several technologies offer the potential to decarbonize the heavy-duty truck sector, including battery-electric trucks and fuel cell vehicles, when paired with a rapidly decarbonizing grid.^4−6^ Multiple major truck manufacturers have announced new battery-electric truck models,^7−9^ driven in large part by lithium-ion battery capacity improvements and cost reductions.^10−12^ In the U.S., some states are passing ambitious policies related to truck electrification. For example, California aims to achieve a full transition to zero-emission drayage trucks by 2035 and a transition to 100% zero-emission medium- and heavy-duty trucks on the road by 2045.^13^

**Figure 1:**
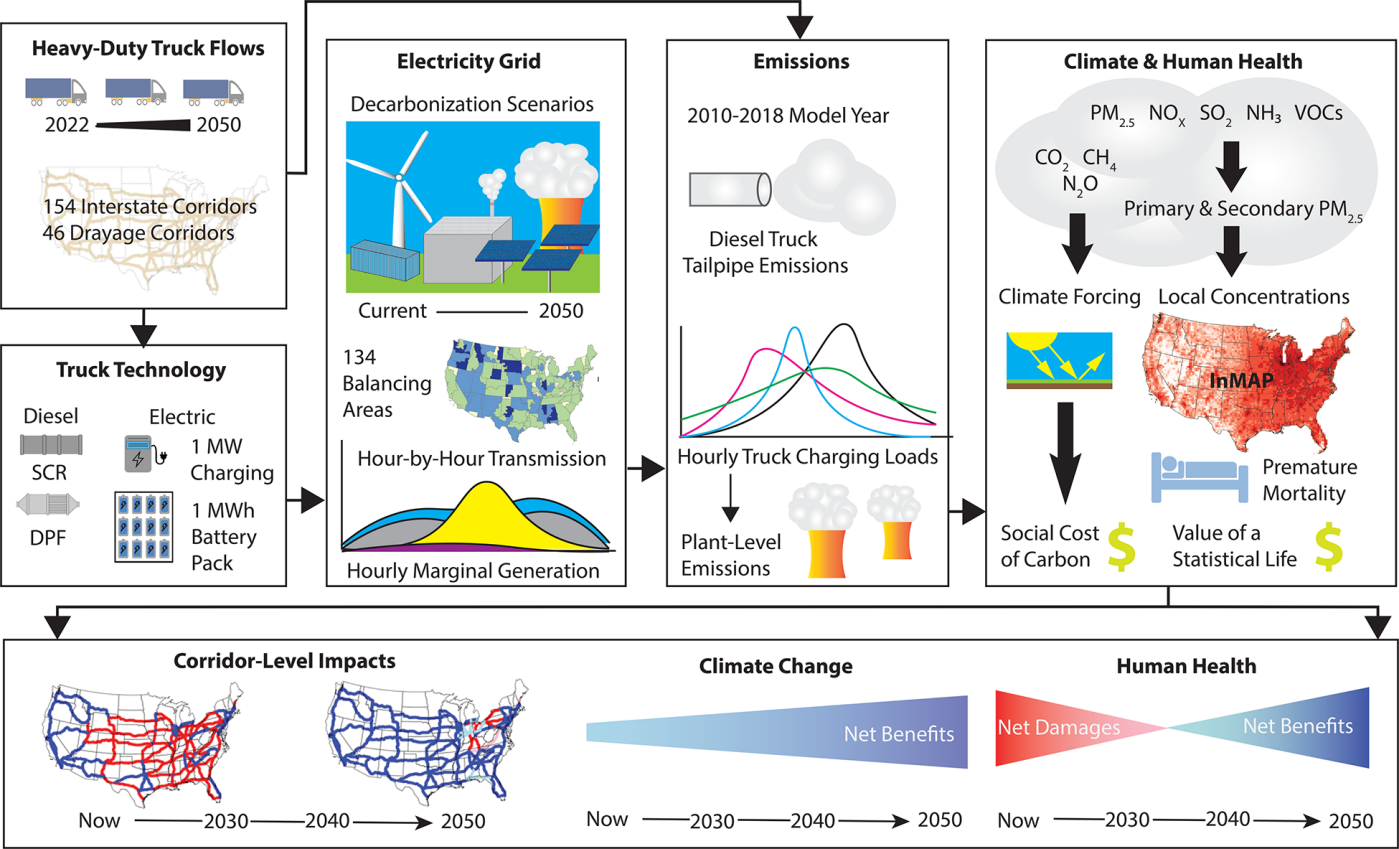
A framework for a corridor analysis that compares health and climate damages between heavy-duty diesel and electric trucks along major U.S. freight corridors, with a focus on the evolving electricity grid through 2050.

To develop the infrastructure needed to support freight electrification, the Federal government is taking a corridor-by-corridor approach. In February 2023, the Department of Energy announced funding to lay the groundwork for charging infrastructure along specific freight corridors and regions in the U.S. including I-95 from Georgia to New Jersey, Northeast freight corridors, the San Francisco Bay Area, and the greater Salt Lake City region.^14^ However, under the current business-as-usual grid mix, electrifying some corridors could increase health and climate damages due to the induced increase in generation from fossil fuel power plants to meet charging loads.^3,15,16^ The question is *when* it will become beneficial to electrify major freight corridors and how those timelines may align with infrastructure build-out. This study uses heavy-duty truck flows, simulated charging loads across 134 regional grid balancing areas (including power flow between balancing areas), future grid scenarios, and integrated assessment modeling to answer that question (Figure 1). Trends in renewable electricity generation costs have a substantial impact on the results; if the cost of renewables is low, it will be net beneficial from an air pollution standpoint to electrify most of the country’s 200 corridors by 2040. However, the near-term effects of low-cost renewables can cause counterintuitive results during the transition period, where emissions-intensive coal power will temporarily satisfy a portion of marginal electricity demand in some regions.^17^

By redirecting the research question from *whether* freight trucks should be electrified to *when*, this study provides actionable information to inform charging infrastructure build-out priorities and company decisions to electrify their fleets based on the most commonly used routes. We compare corridor-specific effects of diesel vs electric trucks through 2050 with three different goals in mind for long-haul and drayage trucking: (1) reducing local/regional air pollution-related health impacts, (2) reducing climate impacts, and (3) reducing the sum of monetized health and climate damages. By exploring monetized damages, this work also illustrates the effect of different social cost of carbon values on the trade-offs between health and climate impacts. We compare monetary damages under the Federal government’s interim social cost of carbon ($51/tonne)^18^ to the recently proposed value of $190/tonne^19,20^ to elucidate how the weight placed on climate impacts versus air pollution may impact decision-making. Although this study focuses on battery electric trucks, a similar corridor-level and year-by-year analytical approach could be used to prioritize hydrogen generation and fueling investments or even compare battery-electric with hydrogen fuel cell trucks.

## Materials and Methods

We analyzed how differences in energy systems across the United States would result in varying quantities of air pollutant emissions, and therefore health impacts, from heavy-duty (i.e., Class 8, GVWR > 33,000 lbs) truck electrification. To quantify these changes, we developed an integrated assessment framework for a corridor analysis that compares health and climate damages between heavy-duty diesel and electric trucks along major United States freight corridors (Figure 1, “Corridor-Level Impacts”). We consider changes in energy systems over time to predict how these health and climate impacts will change between now and 2050 (Figure 1, “Electricity Grid”). To the best of our knowledge, this framework is the first study to examine the health and climate impacts of freight electrification at a national scale while maintaining regional heterogeneity through isolating the impacts of individual corridors (Figure 1, “Climate and Human Health”). By looking at the change over time, we hope this study will be used to guide future long-haul truck electrification efforts by showing how the health and climate impacts vary by location.

### Truck Flow Model and Electric Truck Parameters

We modeled truck flows for the 200 major freight corridors selected by Tong et al.,^3^ which include 154 interstate corridors as well as 46 drayage (i.e., short-haul, intermodal freight) corridors. An origin-destination database, derived from the Freight Analysis Framework’s (FAF) highway assignment database,^21^ was used for truck flows as described in Tong et al.^3,22^ FAF^21^ was also used to project increases in truck flows through 2050. Detailed methods on how truck flows and future projections were incorporated into this model are discussed in the “Truck Flows” section of SI. While there are over 164,000 miles of highway in the U.S.,^23^ our model considers only 29,945 highway miles. This highway network allowed us to study inter-regional freight and drayage transport; however, it may not capture the full extent of freight trucking damages. Future studies could benefit from more coverage across the full highway network and both heavy- and medium-duty trucks, although this is more challenging due to limited availability of data on short-haul and medium-duty truck routing and volumes.

Because the timing of future truck design and battery improvements is uncertain, we held truck design parameters constant from 2022 to 2050, allowing only truck flows and the electricity grid to change over time. This allowed us to isolate the impacts of shifts in electricity generation and emissions. Battery pack capacity was set at 1 MWh. The battery that powers heavy-duty electric trucks can add significant weight to the vehicle, affecting the payload when subject to gross vehicle weight limits. Details of how our model considers the effects of added battery weight is discussed in the SI. Results presented in the main text assumed a base-case battery pack specific energy of 240 Wh/kg. However, battery technology improvements could increase this energy density. To capture the effects of this change, we explore our results under two different battery scenarios in Table S6 in the SI: base-case (240 Wh/kg) and optimistic (320 Wh/kg) battery pack specific energy. Tong et al.^3^ showed that charging power did not significantly affect health and climate damages. However, for the purposes of this study, the charging power was held at 1 MW.

### Charging Load Profile Model

Following the methods of Sripad and Viswanathan^24^ and Tong et al.,^25^ a vehicle powertrain model was used to determine truck energy consumption along each corridor. This model considers how truck speed, truck weight, and road grade impact energy consumption. Using truck flows and energy consumption, we employ a fairly simple strategy for locating hypothetical charging stations and assigning charging loads to each station, consistent with the work of Tong et al.^3^ Evenly spaced stations were placed along highway intersections and as needed throughout each corridor in a way that ensured sufficient infrastructure for supporting long-haul trucking flows.

Previous long-haul truck electrification studies have aggregated charging loads at a state or regional level (e.g., North American Electric Reliability Corporation [NERC] regions),^3,25^ which is a commonly used approach but potentially results in an oversimplification of power flows by assuming that all plants respond equally to increased load regardless of where in the NERC region that load occurs. To capture likely flows of power in response to marginal increases in load, this study maps charging loads to balancing areas. Balancing areas split the U.S. into 134 different county aggregates that approximately balance electricity supply and demand, with power flowing across boundaries as needed.^26^ Using this balancing area-level approach, we are able to integrate charging loads with future grid scenarios.

### Electricity Grid Model

One of the greatest challenges in quantifying the health and climate impacts of vehicle electrification is predicting future power plant emissions and locations, particularly several decades into the future. While regression-based approaches can offer arguably better predictions of marginal emissions in response to changes in load, these models are suited only for short-term scenarios and marginal changes of total load.^27−29^ For longer-term scenarios, a combination of capacity expansion and dispatch modeling is required to predict future generation mixes and emissions with and without vehicle charging loads. To accomplish this, we leverage the NREL Standard Scenarios, which are a set of projections for how the U.S. energy system will evolve over time under multiple pathways and future scenarios.^30^ Our model aggregates hourly charging loads to each of the 134 balancing areas in the contiguous U.S. and assigns generation from individual power plants to truck charging loads based on marginal generator types and locations on an hourly basis.

While there has been much debate over the correct way to model the U.S. electricity grid, Ryan et al.^31^ provided a set of recommendations for selecting the appropriate grid model under different circumstances. When modeling incremental changes in demand, such as the initial penetration of electric trucks into the existing fleet, a short-run marginal grid model is recommended.^31^ This is enhanced by the fact that a short-run marginal grid model can consider power transmission between balancing areas,^32^ a caveat that can be quite important given that we are considering spatial heterogeneity in our corridor analysis. When loadings and generators are modeled at the balancing area level, power is still allowed to flow between balancing areas when needed. In each hour, balancing areas can share marginal generators, forming transmission connected regions (T-regions).^32^

Electricity load in each T-region is allocated to all currently operating individual power plants in that T-region that are classified as the marginal generator type (e.g., natural gas combined cycle, coal, wind). For example, for an hour (and T-region) where natural gas combined cycle (NGCC) is classified as the marginal generator, all currently operating NGCC power plants would be ramped up proportionally to their current generation^33^ until the additional load is met. This means that larger plants provide a greater fraction of additional generation. The approach described here is imperfect, as some currently operating power plants will be decommissioned in the future, and new facilities may be built in new locations. In reality, remaining emissions from fossil-fuel-fired power plants are likely to be concentrated in fewer locations where fossil-fuel-fired power plants remain, but the location of those remaining plants is uncertain.

We studied changes in electricity demand for several decades, up to 2050, across multiple scenarios representing different levels of renewables penetration in the U.S. These renewable energy pathways are outlined in NREL’s Standard Scenarios.^30,34^ Our model has the capability to run results with NREL’s high renewable energy cost, low renewable energy cost, and midcase renewable energy cost scenarios. More information on these scenarios can be found in NREL’s Scenario Viewer^34^ and “2022 Standard Scenarios Report: A U.S. Electricity Sector Outlook”.^30^

### Diesel Truck Parameters and Emissions

For consistency across scenarios and years, a single diesel truck model is selected for comparison against electric trucks. The diesel truck model selected was Model Year (MY) 2010–2018, which contains Diesel Particulate Filter (DPF) and Selective Catalytic Reduction (SCR) technology, representing a dramatic reduction in air pollutant emissions relative to earlier models.^3^ Our decision to focus on a single diesel truck model allowed us to isolate the impacts of an evolving electricity grid across time and new policy. However, a single truck model is unlikely to represent all newly purchased diesel trucks over several decades.^35^ Tong et al.^3^ showed that beyond MY 2010–2018 trucks with DPF and SCR, only minor changes to air pollution emissions can be expected. While this is only an incremental change, new models that study future diesel truck fleets may want to consider this factor in addition to an evolving electricity grid. A comparison of damages under base-case vs future truck models is explored in Table S7 in the SI. Assuming trucks remain in operation for approximately 15 years and future tailpipe emissions reductions for newer trucks will be small, our selected model year should be reasonably representative of both the typical new truck purchase and the overall diesel Class 8 fleet into the foreseeable future. Tailpipe emission factors were based on the GREET model^36^ and on-road measurements from Preble et al.^37^ These on-road measurements were fleet-averaged for specific model years and include superemitters.^37^ More details on both diesel and power plant emission factors can be found in the “Emission Factors” section of the SI.

### Health and Climate Impacts

We estimated the health and climate impacts associated with heavy-duty freight trucking on each of the 200 major corridors in the U.S. for an entirely diesel fleet and an entirely electrified fleet. We then compared these diesel and electric truck impacts to determine where electrification of some or all of the fleet would yield net climate, health, and monetized benefits when climate and health damages are summed. We made these comparisons in four different years: 2022, 2030, 2040, and 2050. The only change across each of those years is the composition of the grid and the resulting emissions. We did not attempt to adjust the geographic distribution of population with time.

All air-pollutant-related health damages quantified in this paper are based on primary and secondary fine particulate matter (PM_2.5_). PM_2.5_ is one of the highest mortality risk factors in the 21st century, responsible for the majority of deaths associated with air pollution exposure.^38^ PM_2.5_ pollution is associated with diesel trucks, both through direct emissions and through secondary formation from NO_*x*_.^39^ While diesel trucks are still major emitters of PM_2.5_ and NO_*x*_, the widespread adoption of SCR and DPF has reduced these emissions on a per-kilometer basis considerably.^40,41^ From a life-cycle perspective, electric trucks increase greenhouse gas (GHG) and air pollutant emissions through the electricity generation needed to meet charging demand in addition to upstream emissions associated with battery and vehicle production. Because electricity demand can be met from power plants outside of the region where the demand occurs^32^ and health effects of electricity generation can be found far from the actual emissions source,^42^ the geospatial extent of damages from electricity generation can be greater for electric trucks than for diesel.

The health impacts of long-haul trucking are determined through changes in PM_2.5_ concentrations due to either diesel trucks or electric trucks. These PM_2.5_ changes were found using a reduced-complexity air quality model, the InMAP source–receptor matrix (ISRM).^42−44^ This matrix relates emissions to air quality in specific locations, estimating the change in PM_2.5_ concentrations considering both primary emissions as well as secondary formation from relevant precursors.^44^ Each grid cell has an associated population, which allowed us to quantify health impacts in the form of mortality associated with changes in PM_2.5_ concentrations.^44^ We estimated changes in PM_2.5_ concentrations from each long-haul truck corridor due to tailpipe emissions from diesel trucks compared with power plant emissions from electric trucks. We then translated these concentrations to expected changes in mortality attributable to the truck travel on each corridor following the methods of Krewski et al.^45^ and Tessum et al.^44^

### Battery Manufacturing and Upstream Emissions

We estimated monetary health and climate damages from upstream impacts for diesel and electric trucks, including battery manufacturing and resource extraction, using data reported in the literature.^3,46−48^ Our assessment of impacts from battery manufacturing considers battery capacity, electric truck lifetime, and battery lifetime. Given that battery technology is evolving rapidly,^49,50^ battery manufacturing emissions are especially uncertain when projecting changes through 2050. This is compounded by the fact that load shapes for battery manufacturing facilities are not well documented, nor is it clear where the facilities will be built. To minimize uncertainties, our main-text results and corridor-by-corridor analysis focus specifically on the use-phase of diesel vs electric trucks. The underlying assumption is that upstream emissions will not vary based on which corridor is being electrified. Total health and climate impacts, including from battery manufacturing and other upstream sources, can be found in Table S1 of the SI. More information about the upstream emissions calculation can be found in the “Battery Manufacturing and Upstream Emissions” section of the SI. We assume that these upstream impacts will hold through 2050. However, battery improvements and the location of manufacturing facilities are highly uncertain.^51^ If improvements are made to battery technology, this would further improve the benefits of heavy-duty truck electrification. As updated upstream impacts are released in the literature, this analysis should be updated to reflect the current values.

### Corridor Analysis

We determined which trucking corridors are beneficial to electrify year-by-year compared to diesel trucks based on three different criteria: (1) net impacts on air pollution-related premature mortality, (2) net greenhouse gas emissions (on a 100-year global warming potential basis), and (3) the sum of net changes to monetized health and climate damages. All health and climate impacts originating from trucks operating on a given corridor are allocated to the corridor on which they drive, even if the actual health damages occur in communities located far from the corridor. If electric trucks operating in a given corridor result in a net decrease in premature mortality or GHG emissions compared to diesel trucks (using model year 2010–2018 emission factors and efficiency), they are categorized as resulting in a net benefit. We also quantify the sum of health and climate damages by using the value of a statistical life and social cost of carbon to convert mortality and GHG emissions into a single net monetary cost relative to diesel trucks.^18,19,43^ Given the ongoing discourse regarding the appropriate social cost of carbon, we consider two values ($51 and $190/tonne) in our analysis.^18−20^ This analysis shows how the social cost of carbon could affect which freight corridors are beneficial to electrify.

## Results and Discussion

### Corridor Analysis of Health Impacts

**Figure 2:**
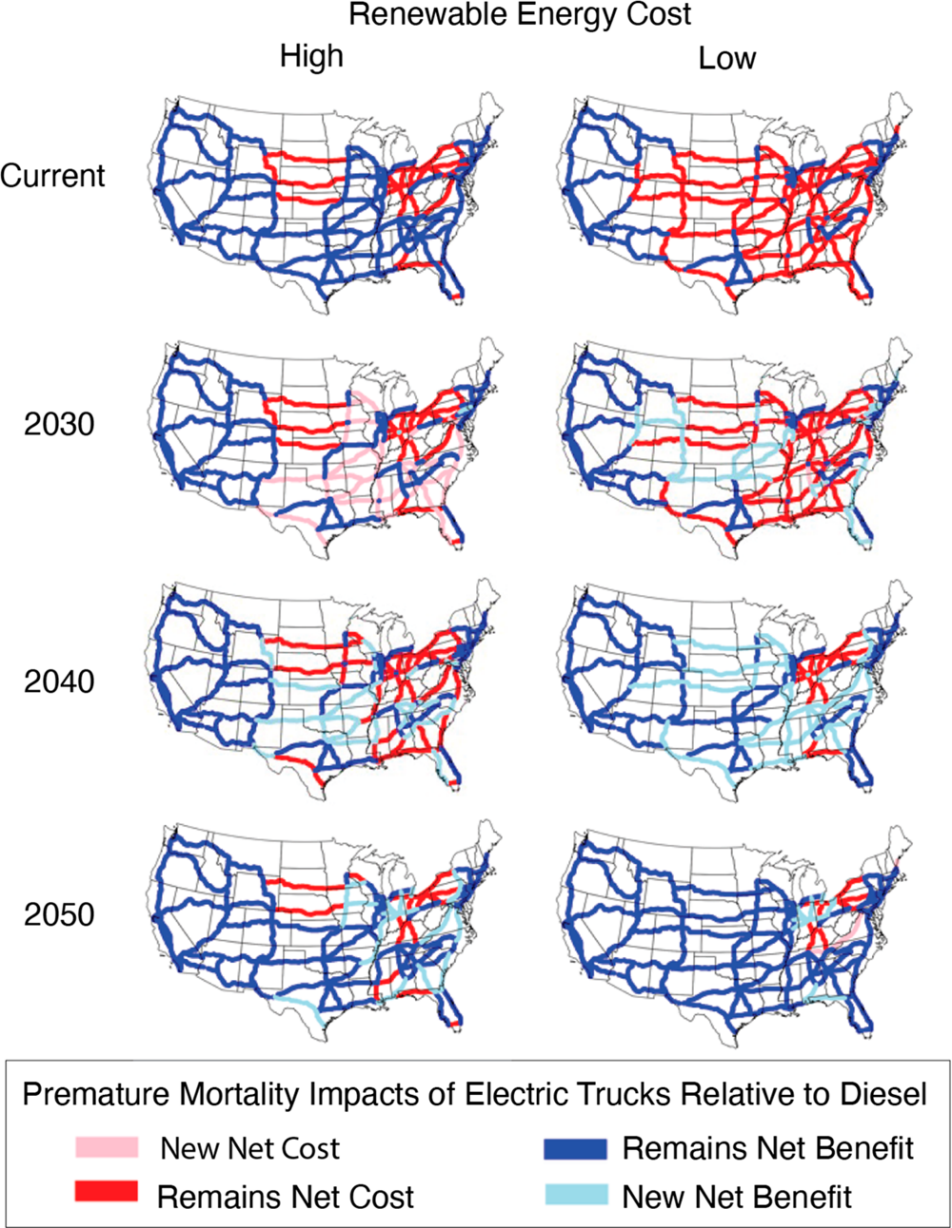
Corridor analysis showing which corridors are beneficial to electrify for reducing air-pollutant-related mortality compared to diesel trucks. These health damages include mortality from primary PM_2.5_ emissions, as well as secondary formation. We show how beneficial corridors change over time as regional electricity grid mixes change. Two electricity grid scenarios are shown: (1) low renewable energy cost (right column) and (2) high renewable energy cost (left column). “New Net Benefit/Cost” and “Remains Net Benefit/Cost” are relative to the previous decade (and image) shown. The “Current” year is the first year analyzed, so this image simply shows “Net Benefit” vs “Net Cost”.

The degree to which electrifying long-haul and drayage trucks impact human health and climate forcing relative to a diesel truck baseline is largely dependent on the share of renewable energy on the electricity grid. For this reason, we consider multiple scenarios that reflect uncertainty in renewable energy costs. The cost of renewable energy affects the number of long-haul truck corridors that are beneficial to electrify on the basis of air pollution-related human health impacts and in what year they become beneficial (Figure 2). While this analysis shows the number of corridors with net benefits, results for additional metrics (i.e., share of VMT and road miles with net benefits) are included in Tables S4 and S5 of the SI. Similarly, Figure S2 in the SI provides additional detail by showing the percentage increase or decrease in premature mortality for each corridor resulting from a switch from diesel trucks to electric trucks.

In the near-term, a low renewable energy cost scenario means that most corridors are not favorable to electrify from a human health standpoint. This counterintuitive result has been documented in other studies and observed in real-world hourly grid mixes;^3,27,28^ the effect is driven by the fact that rapid deployment of renewables places coal on the margin in the near-term. While the curtailed use of coal-fired generation is a positive development for air quality (increased penetration of renewables is displacing coal), it also means that any short-term, nonmarginal increase in load is likely met by those same coal plants. This effect is only captured in studies that focus on the marginal impacts of electrification as opposed to using the average grid mix. In contrast, when the near-term cost of renewable energy is high, many regions rely on cleaner, natural-gas-fired power plants. This is expected to change markedly between the current year and 2050. By 2030, as more renewables come online, additional corridors will be beneficial to electrify under a low renewable energy cost scenario compared to a high renewable energy cost scenario. However, the biggest change occurs between 2030 and 2040 when most corridors result in net reductions in air pollution-related mortality if trucks are electrified. This is particularly true in low renewable energy cost scenarios. In 2040 and 2050, there are only a few corridors that remain unfavorable to electrify from a human health standpoint, where wind and solar resources are more limited and fossil energy reliance persists, even in 2050. Figure S4 in the SI shows in greater detail which energy sources act as the marginal generator for the different scenarios and how this changes over time.

Table 1 provides the national-level context for our results by showing net changes in premature mortality resulting from full electrification of trucks along all 200 corridors under multiple grid scenarios compared to 2010–2018 model year diesel trucks (which are likely representative of current and future diesel trucks on an emissions basis). Note that mortality from diesel trucks increases each decade due to increasing truck flows over time (see SI for further details). Mirroring the corridor-by-corridor results, Table 1 indicates that low renewable energy costs translate to higher near-term air pollution-related health impacts when marginal grid emissions are attributed to electric trucks. By 2040, low renewable energy costs would translate to a 13% reduction in national-level health burdens if trucks are electrified. In 2050, all scenarios offer a reduction in health damages, ranging from 27% to 38%. One may note that the total number of premature deaths across all scenarios (507 to 824 per year) is relatively small compared to other causes of death (e.g., traffic fatalities). However, premature mortalities are an incomplete metric to capture the full health burden of air pollution, which contributes to asthma and other morbidities that are not included in InMAP and other comparable models.^42,43,52,53^ While studies have shown that the majority of monetized damages related to air pollution is caused by premature mortalities,^52^ this method helps to identify geographic disparities in the health effects of heavy-duty trucking.

**Table 1:** Comparison of Total Air Pollution-Related Premature Mortality, Measured in Total Deaths, Across All Corridors for Diesel Trucks and Electric Trucks^a^

		Grid Scenario: Renewable Energy Cost		
Year	MY 2010–2018 Diesel Trucks	Low	Mid-Case	High
Near Future	568	788	825	580
2030	630	780	803	780
2040	710	**616**	761	750
2050	824	**507**	**556**	**601**

a Bold entries indicate a net reduction in damage relative to diesel trucks.

### Health Impacts of Interstate and Drayage Trucks

Although this study includes both interstate corridors and drayage (i.e., short-haul, intermodal freight) trucks, the rollout of electric trucks on these types of corridors is likely to be different. Long-haul (also referred to as line haul) trucking relies on new trucks, and these remain in service for 3–5 years before being sold to regional carriers. Drayage, in contrast, relies on older trucks that are no longer suitable for longer-haul routes. Their trips are shorter, they operate primarily in urban areas, and their air pollutant emissions disproportionately affect disadvantaged communities.^54−57^ Therefore, a relevant question is whether there is a substantial difference in the health impacts of electrifying drayage versus interstate corridors. Table S3 in the SI shows that drayage trucking corridors are more beneficial to electrify compared to interstate trucks on the basis of health impacts alone. Of the 200 truck corridors considered in this analysis, 76–91% of drayage corridors are beneficial to electrify from a health standpoint in the near-future compared to 20–68% of interstate corridors. By 2050, 98% of drayage truck corridors are beneficial to electrify for all scenarios compared to 66–86% of interstate corridors. This is due to the fact that densely populated urban areas tend to have more port and rail activity and therefore more drayage trucks.^58,59^ Additionally, in urban areas, diesel trucks tend to have a higher intake fraction of emitted pollutants compared to electric trucks due to close proximity between emissions sources (i.e., roadways) and communities.^57^

### Corridor-Level Greenhouse Gas Emissions

**Figure 3:**
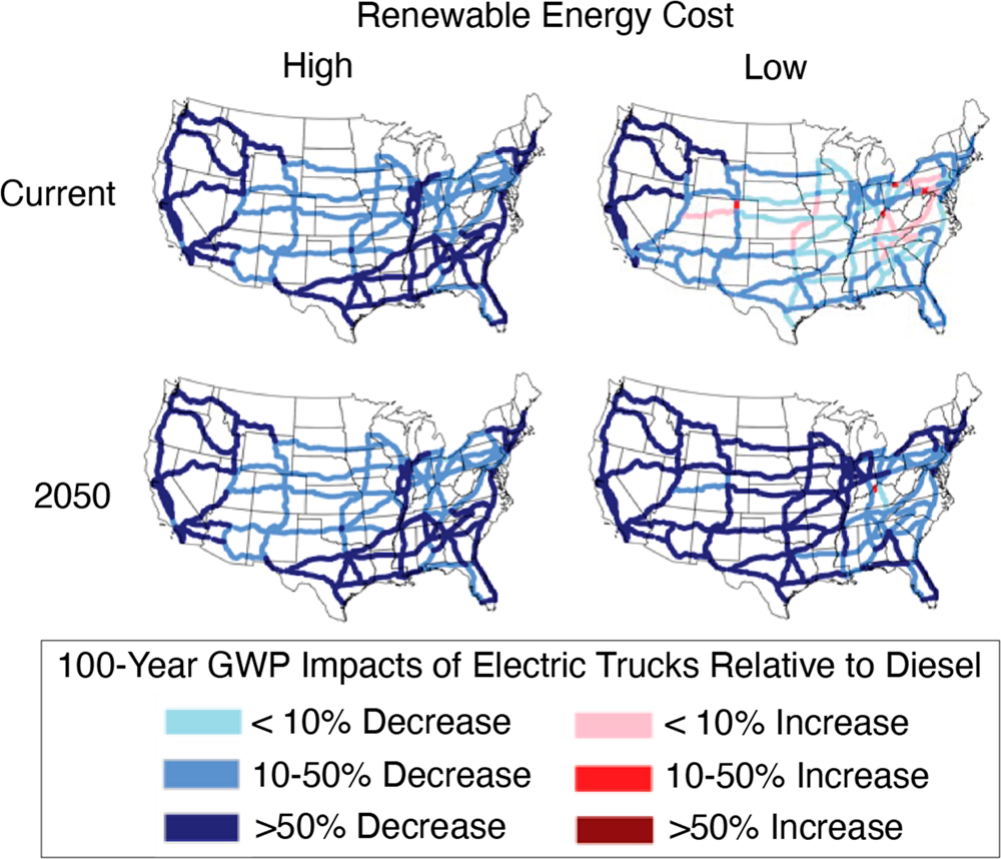
Corridor analysis showing the percentage change in greenhouse gas emissions over time for the switch to electric trucks compared to the base-case of diesel trucks. Two electricity grid scenarios are shown: (1) low renewable energy cost (right column) and (2) high renewable energy cost (left column).

The greenhouse gas implications of truck electrification are more straightforward to assess. Most corridors are beneficial to electrify under the present-day conditions for a low renewable energy cost scenario (Figure 3). By 2050, nearly every corridor will see a decrease in GHG emissions with the switch from diesel to battery-electric trucks. Most of the 2050 corridors under a low renewable energy cost show a greater than 50% decrease in GHG emissions compared to diesel trucks. Under high renewable energy costs, every corridor is beneficial to electrify currently with many corridors showing a greater than 50% decrease in GHG emissions compared to diesel trucks.

### Monetary Health and Climate Damages

**Figure 4:**
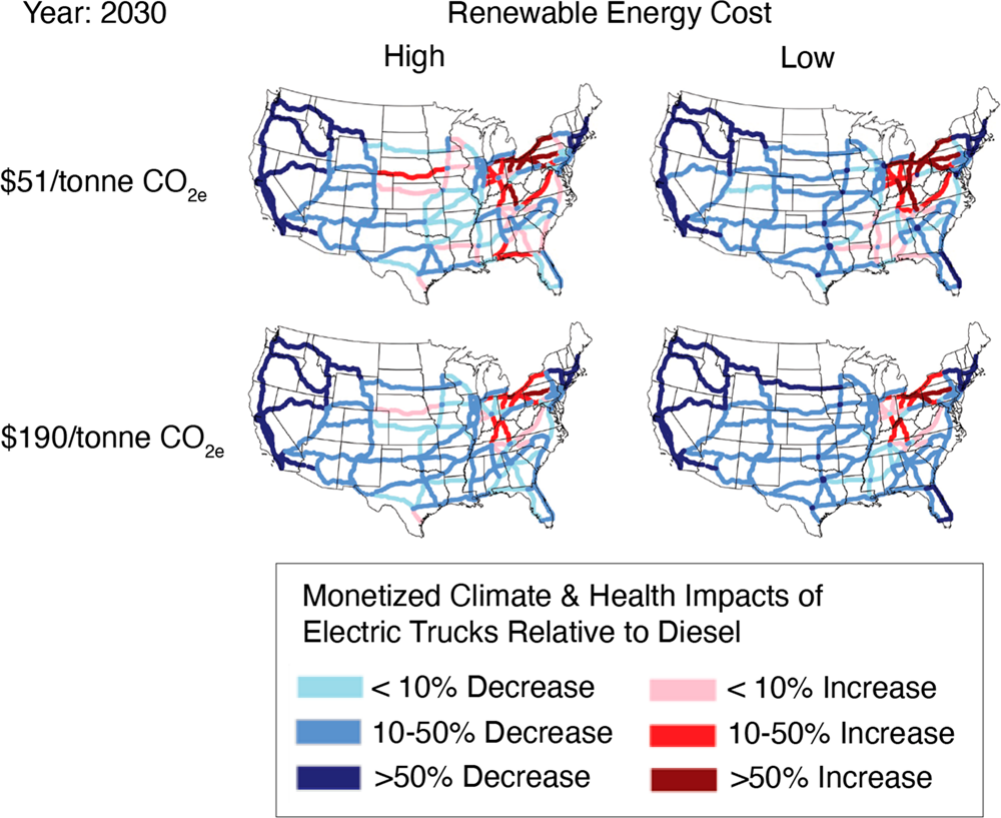
Corridor analysis for 2030 showing percentage change in monetary health and climate damages over time for the switch to electric trucks compared to the base-case of diesel trucks. We compare how the results change under two different social cost of carbon values: (1) $51/metric ton (top row) and (2) $190/metric ton (bottom row). Two electricity grid scenarios are shown: (1) low renewable energy cost (right column), and (2) high renewable energy cost (left column).

On many corridors, electrifying trucks reduce GHG emissions sooner than it reduces human health burdens from air pollution. The weight placed on GHG emissions through the social cost of carbon has a direct impact on the number of corridors that are beneficial to electrify with regard to reducing the monetary health and climate damages of diesel trucks. Figure 4 shows how two different values for the social cost of carbon impact the net costs or benefits of electrifying trucks in 2030. Under all renewable energy cost scenarios, when the higher social cost of carbon is used ($190/tonne of CO_2e_), more corridors are beneficial to electrify. This is consistent with findings in Figure 3 suggesting that even corridors that are associated with net health burdens result in net GHG benefits.

### Impact of the Inflation Reduction Act

**Figure 5:**
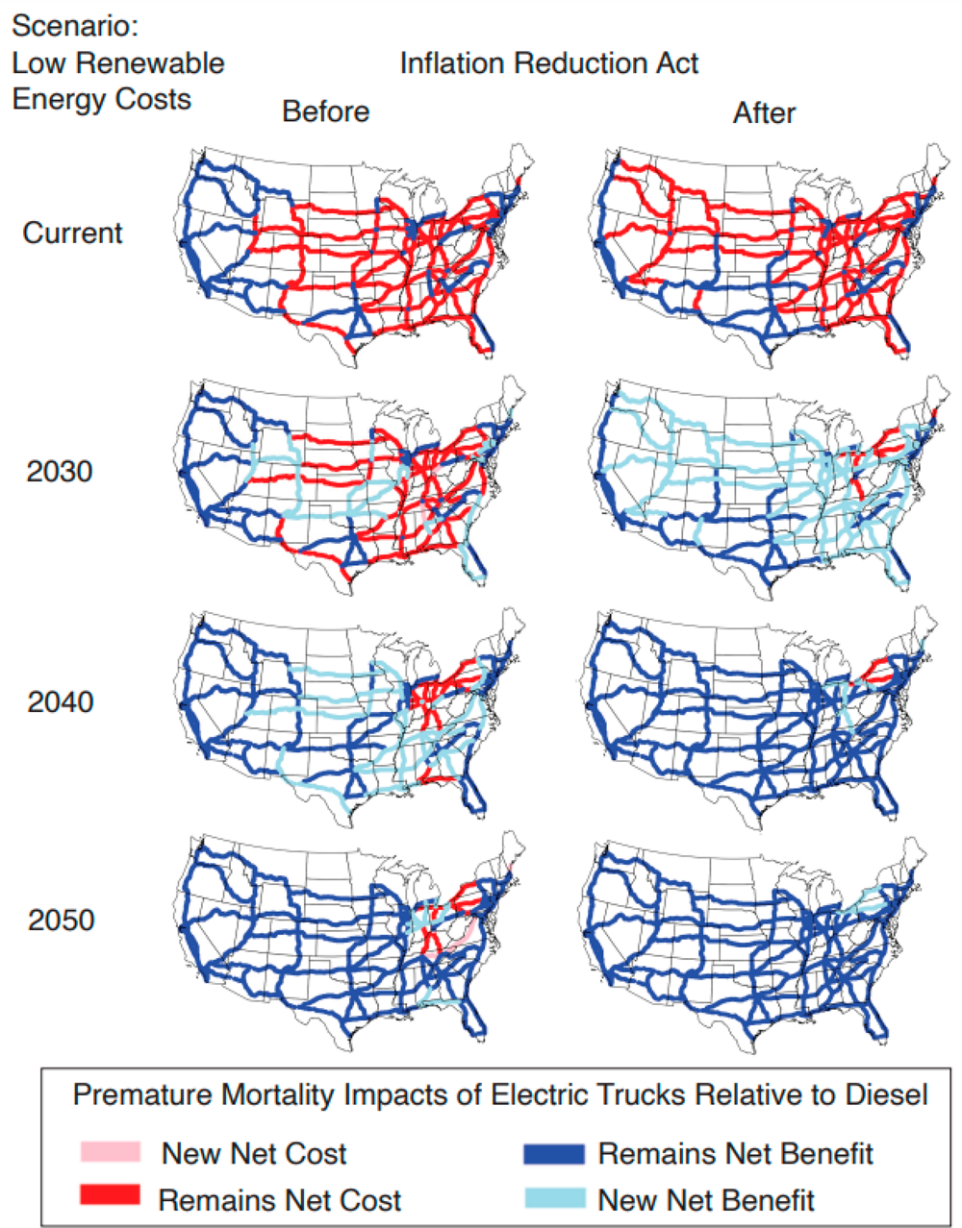
Corridor analysis showing which corridors are beneficial to electrify for reducing air-pollutant-related mortality compared to diesel trucks with and without the Inflation Reduction Act. These health damages include mortality from primary PM_2.5_ emissions as well as secondary formation from relevant precursors. We show how beneficial corridors change over time as regional electricity grid mixes change. Only one electricity grid scenario is shown: low renewable energy cost. “New Net Benefit/Cost” and “Remains Net Benefit/Cost” are relative to the previous decade (and image) shown. The “Current” year is the first year analyzed, so this image simply shows “Net Benefit” vs “Net Cost”.

The Inflation Reduction Act of 2022 is a recent US policy that offers a variety of tax credits and grants to support renewable energy development and the construction of transmission infrastructure.^60^ So far, this article has presented results without incorporating the effects of the Inflation Reduction Act (IRA), in part because the full effects of this recent policy development are still being analyzed. However, in 2023, the National Renewable Energy Laboratory released updated Standard Scenarios for 2022,^30^ which account for tax credits and other provisions in the IRA that will affect the electricity grid mix and generator dispatch through 2050. To understand how the IRA may impact our conclusions, we reran the analysis with those updated scenarios, which are directly comparable to our prior outputs. The updated results are striking. Under a low renewable energy cost scenario, by 2030, 128 corridors are beneficial to electrify without the IRA, and with the IRA, this number increases to 188 corridors (Figure 5). This is because by 2030, renewable energy sources are expected to replace natural gas as the most frequently occurring marginal generator. Coal, in particular, is expected to sharply decline as the marginal generator choice post-IRA. This result is illustrated in greater detail in Figure S5 in the SI. Table S2 in the SI shows total air pollution-related premature mortality from full truck electrification in the U.S. before and after the IRA.

### Limitations and Future Work

For consistency across years and scenarios, several key factors were kept constant in order to determine the effects of a changing grid on the environmental trade-offs of electrifying heavy-duty trucks. However, battery technology changes will likely have major implications for health and climate impacts over time,^49,50^ and manufacturers will be searching for more cost- and energy-efficient strategies for electrifying trucks as the industry matures. Additionally, future improvements in diesel truck and power plant efficiency and emissions control technologies may affect GHG and air pollutant emissions in ways that are not captured in this study.^40^ For example, if fossil-fueled power plants make use of carbon capture and sequestration, the air pollutant emissions profile will be very different from power plants today, with reductions in some pollutants and increases in ammonia.^61^

The cost of individual electric trucks, as well as the charging infrastructure required for their operation, will be a major driving factor in the adoption of this technology. Future work should conduct a benefit-cost analysis that considers the full cost of truck electrification. This analysis should include the social cost of electric and diesel trucks, considering greenhouse gas emissions and monetized health effects. Additionally, the possibility of modal shifting should be considered. While freight projections through 2050 were determined by the Freight Analysis Framework, which considers 8 domestic freight modes and 7 international modes,^21^ other modes of transportation (i.e., trains) may be more suitable for electrification and could warrant mode shifting. While studies have examined the cost and greenhouse gas reductions of mode shifting internationally^62−64^ (e.g., Europe and Canada), limited research is available on applications for the U.S. Future work should compare the costs and emissions between truck and train electrification in the U.S. and consider the possibility of mode shifting.

The causal linkage between emissions and human health impacts is another source of uncertainty. Using the ISRM to estimate health impacts from primary and secondary particulate matter enables us to run multiple grid scenarios and years in a fraction of the time it would take a traditional air quality model to achieve results.^44^ However, reduced-form air quality models have limitations. Over time, population distribution may change^65^ (e.g., more people moving from rural to urban areas), potentially impacting the air pollution-related mortality caused by the corridors considered in this study. Variations in the chemical composition of particulate matter can have different health impacts. Of particular importance to this study, PM from diesel can be carcinogenic, causing more health effects than many other forms of PM.^66,67^ Additionally, we are projecting changes in health effects from now until 2050. The ISRM calculation relies on existing concentrations of pollutants as well as current estimates of mortality rates.^44^ Over the next several decades, these concentrations and mortality rates may change, affecting the atmospheric processes that lead to secondary PM formation^68−70^ and associated health effects.^71,72^ Future studies may benefit from analyses to understand how shifting background concentrations may impact their results.

### Grid Model Selection

There has been much debate over the correct way to model the electricity grid,^16,28,73,74^ and although this study is focused on marginal emission rates, we acknowledge that this debate is not settled in the energy and emissions modeling community. Marginal emission rates have been used by several studies to predict electricity grid emissions.^27,28,75−77^ Holland et al.^28^ argue that a marginal grid model is the correct method for modeling GHG emissions in the United States, pointing to prior studies that show that marginal emission factors more accurately predict electric vehicle emissions over average emission factors.^31,77−79^ Gagnon et al.^74^ argues that short-run marginal emission rates do not consider the fact that large, persistent changes in demand can structurally change the electricity system, impacting emissions. This is the basis for long-run marginal emission rates.^74^ On the other hand, Lin^16^ argued that an average electricity generation (AEG) approach should be used over marginal for electric trucks due to the long-term change in demand. This is in contrast to several other studies which say that a marginal grid model is suitable for predicting emissions from electric vehicles and should be selected over AEG.^28,31,77−79^ Ryan et al.^31^ compared several electricity grid emissions models and provided a set of recommendations for selecting the appropriate model under different circumstances. When modeling an incremental demand, like the penetration of electric trucks into the existing fleet, marginal emission factors are recommended.^31^ The manner and time scale in which independent system operators and regional transmission organizations adjust their planning to accommodate electrified trucks will impact the causal linkage between charging loads and marginal changes in power plant emissions.

### Implications for Future Infrastructure Investments

The encouraging results on the effect of the Inflation Reduction Act shown in Figure 5 suggest that near-term investments in freight truck charging infrastructure are warranted to ensure that most trucks are electrified within the 2030 to 2040 time frame. In particular, our results indicate that infrastructure and incentives that accelerate electrification of trucks on drayage corridors can yield near-term benefits for human health and the climate. The Inflation Reduction Act appears to play a substantial role in accelerating the transition to net air pollution benefits across most corridors. Effects of the IRA are most notable in the South, Mid-Atlantic, and parts of the Midwest, where renewable resources are more limited, and policy supports enable a faster transition away from high-emitting fossil fuel power plants. Applying this corridor and time-dependent approach to evaluating electrification impacts can enable more strategic rollout of infrastructure and a better understanding of the interplay between technology and policy.

## Supporting Information Available

Additional methodological assumptions including battery technology, electricity transmission, emission factors, and upstream and life-cycle impacts. Full results are presented including mortality and greenhouse gas emissions from each individual corridor, a list of corridors, and nationwide mortality under a variety of truck and battery improvement scenarios (PDF)
