# Supplementary material for: Corridor-Level Impacts of Battery-Electric Heavy-Duty Trucks and the Effects of Policy in the United States
Source: Environ Sci Technol. 2023 Dec 18;58(1):33–42. doi: 10.1021/acs.est.3c05139 (PMC10785805; doi:10.1021/acs.est.3c05139)
Supplement: Supplementary file 1 — es3c05139_si_001.pdf [file es3c05139_si_001.pdf]

Supporting Information

Corridor-Level Impacts of Battery-Electric Heavy-Duty Trucks and the Effects of Policy in the United States

Wilson McNeil [1,2,3], Fan Tong [4,5,11], Robert A. Harley [2], Maximilian Auffhammer [1,6,7], Corinne D. Scown [1,8,9,10]*

[1]Energy Technologies Area, Lawrence Berkeley National Laboratory; Berkeley, CA 94720, USA.

[2]Department of Civil and Environmental Engineering, University of California, Berkeley; Berkeley, CA 94720, USA.

[3]Department of Civil and Natural Resources Engineering, University of Canterbury, Christchurch 8041, New Zealand

[4]School of Economics and Management, Beihang University, Beijing 100191, People's Republic of China

[5]Lab for Low-carbon Intelligent Governance, Beihang University, Beijing 100191, People's Republic of China

[6]Department of Agricultural and Resource Economics, University of California, Berkeley; Berkeley, California 94720, USA.

[7]National Bureau of Economic Research; Cambridge, MA 02138, USA.

[8]Life-Cycle, Economics and Agronomy Division, Joint BioEnergy Institute; Emeryville, CA 94608, USA.

[9]Biosciences Area, Lawrence Berkeley National Laboratory; Berkeley, CA 94720, USA.

[10]Energy and Biosciences Institute, University of California, Berkeley; Berkeley, CA 94720, USA.

[11]Peking University Ordos Research Institute of Energy, Ordos City, Inner Mongolia 017020, People's Republic of China

*Corresponding author. Email: cdscown@lbl.gov

This file includes:

Supplementary text

Figures S1 to S5

Tables S1 to S12

Pages S1 to S28

Methods

Data Sources for Electricity Grid Model

The data for the short-run marginal generators came from NREL's Cambium tool which provides hourly grid mixes and short-run marginal generator data at a balancing area-resolution for a given year, up to 2050.[1] The grid mix and short-run marginal generator for each balancing area change each hour throughout the year. Hourly data is crucial for the present study given that electricity demand from long-haul electric trucks varies hourly. Power plant emission factors were adapted from EPA's Emissions & Generation Resource Integrated Database (eGRID).[2]

Truck Flows

Data for heavy-duty truck flows came from the Freight Analysis Framework (FAF), a data set from the Bureau of Transportation Statistics and Federal Highway Administration.[3] FAF provides flow estimates for various freight types (i.e., rail, ship, trucking) in 2017 and projects how these values will change every five years up to 2050.[3] This data is based on the 2017 Commodity Flow Survey as well as international shipping data.[3] FAF data was used to create an origin-destination database following the methods of Tong et al.[4] While Tong et al.[4] used FAF v4.3, which uses 2012 freight flows, more recent data has been released through FAF v5.4, which uses 2017 freight flows. FAF v5.4 follows the same methods as FAF v4.3 only with updated projections.[3] In order to update our origin-destination database, we found the percent change between 2012 truck flows from FAF v4.3 and 2017-2050 truck flows from FAF v5.4, which we then applied to our database. We did this for each year considered in our study (2022, 2030, 2040, and 2050). This update of our origin-and-destination database provided truck flow estimates (based on increasing flows) for interstate and drayage corridors up to 2050. For the purposes of this study, drayage corridors are defined as short-haul corridors (less than 20 miles) that carry goods between multiple modes of transport (e.g., port, rail).

Electric Truck Design

Truck design parameters were kept consistent from 2022 to 2050 in order to focus on the effect of regional grid mixes and a changing electricity grid over time. Battery pack capacity was

held constant at 1 MWh, which can achieve a driving distance of 365-702 km.[5] Following the methods outlined by Tong et al.,[5,6] battery weight is considered in the truck weight and vehicle powertrain models. Payload is calculated by corridor for both diesel and electric trucks. If the payload of diesel trucks exceeds the payload of electric trucks, our model compensates by adding additional electric truck trips to achieve the same payload as the diesel counterparts. Otherwise, the same volume of diesel and electric trucks are assumed. Tong et al.[5] showed that charging power did not significantly affect health and climate damages. However, for the purposes of this study, charging power was held at 1 MW. Charging efficiency was assumed to be 97%. Truck design has implications for aerodynamics and battery-pack-level specific energy.[5] The truck design was assumed to be current (2020), resulting in a base-case battery design of 240 Wh/kg.[5] However, a comparison against improved truck design is shown in Table S7. Electric trucks were assumed to be confined by a gross vehicle weight regulation, affecting the maximum payload that electric trucks could carry compared to diesel trucks.

T-regions

Balancing areas can share marginal generators, forming transmission connected regions (T-regions).[1] The extent of a T-region changes each hour, spanning anywhere from an individual balancing area to a large portion of the country.[1] The process used for creating T-regions is defined in Cambium's documentation.[1] While this data is not publicly available, neighboring balancing areas with the same short-run marginal generator power plant type and short-run marginal cost (within 5%) were assumed to share a marginal generator. This can occur across numerous balancing areas, creating T-regions that vary for each time step.

Our model outputs annual emissions as well as total generation for each corridor. We calculated the percent increase in total electricity demand expected by the switch to battery-electric trucks in 2022 vs. 2050, shown in Figure S1.

Emission Factors

Based on simulated increases in generation and emissions for individual power plants, we can determine the quantity and location of additional annual emissions expected due to an increase in electricity demand. Emission factors used in this study were sourced from the Grid Optimized Operation Dispatch (GOOD) model.[7] These factors were derived for each power plant

in the U.S. based on plant type and specific plant attributes.[2,7] Assigning additional load to individual power plants is important in determining health impacts due to the relationship between proximity to emissions sources and intake fraction of pollutants. Outputs of the model included annual emissions of NO_X, SO_2, N_2O, CH_4, $PM_{2.5}$, and CO_2. These plant-level pollutant emissions, along with stack height, diameter, velocity, and exhaust gas temperature, are needed to estimate health impacts. Greenhouse gas emissions must only be accounted for on a mass of emissions basis; these were normalized to CO_2-equivalents (CO_{2e}) using the 100-year global warming potential of CO_2, N_2O, and CH_4.[8]

Diesel tailpipe emission factors for 2010 – 2018 model year engines were adapted from the study by Tong et al.[5], who in turn calculated the emission factors based on field measurements by Preble et al.[9], data from GREET[10], and mass balance approaches.[11] Outputs of the model included annual emissions of NO_X, SO_2, N_2O, CH_4, $PM_{2.5}$, NH_3, CO, VOC, and CO_2.

Battery Manufacturing and Upstream Impacts

Health and climate damages from upstream sources and battery manufacturing were determined for both diesel and electric trucks and summed across all corridors. The calculations for these damages were adapted from the methods of Tong et al.[5] Health and climate damages from battery manufacturing, as well as lifetime truck mileage, were determined through recent literature.[5,10,12,13] Climate damages from battery manufacturing were based on a social cost of carbon value of $51/tonne.[14] We found upstream health and climate damages from electricity generation and diesel fuel using factors from Tong et al.[15] Total health and climate damages from all corridors and all sources, including all upstream damages, are shown in Table S1.

Total upstream damages =

$$\sum_{corridor,c} \frac{truck\ fleet\ mileage\ (c)}{truck\ fleet\ mileage\ ovr\ lifetime\ of\ battery\ (c)} \times battery\ capacity\ \times weighted - average\ health\ and\ climate\ damages\ from\ upstream\ sources\ excluding\ battery\ manufacturing$$

Total battery manufacturing damages =

$$\sum_{corridor,c} \frac{truck\ fleet\ mileage\ (c)}{truck\ fleet\ mileage\ ovr\ lifetime\ of\ battery\ (c)} \times battery\ capacity\ \times$$

$$weighted - average\ health\ and\ climate\ damages\ from\ battery\ manufacturing$$

Health and Climate Impacts

We determined air-pollution related mortality following the methods of Krewski et al.[16] and Tessum et al.[17] This method assigns an increase in the mortality rate based on a modeled increase in $PM_{2.5}$ concentrations. Based on the results of Krewski et al.[16], for every 10 μg/m^3 increase in $PM_{2.5}$ concentration, there is a 6% increase in expected mortality rate.

In order to attribute air-pollution mortality to individual corridors, we ran the ISRM for each of the 200 corridors individually. We ran results separately for diesel and electric trucks, and we ran each scenario individually as well. When determining a beneficial corridor to electrify, we directly compared either mortality or greenhouse gas emissions between diesel and electric corridors.

Additional Results

Health Impacts

We created a percentage increase/decrease figure for air pollution-related mortality similar to Figure 3 in the main text (Figure S2).

Climate Impacts

We created a change over time figure for greenhouse gas emissions similar to Figure 2 in the main text (Figure S3).

Marginal Generator Count

Each of the 134 balancing areas has a marginal generator in each hour of a given year.[1] For each balancing area, the marginal generator supplies varying amounts of energy depending on the additional load required in a particular region, making this an imperfect metric. However, by comparing the number of times that different electricity types (e.g., coal, natural gas, renewables) appear on the margin, we can observe patterns of which sources meet additional demand for different scenarios. This helps us understand the variation in emissions, and therefore health and climate impacts, for the scenarios used in the present study. Figure S4 shows the

number of times that three primary electricity types (coal, natural gas, and renewables) act as the marginal generator for any of the balancing areas in a given year under two different scenarios: high renewable energy cost and low renewable energy cost. Figure S5 shows the marginal generator count under a low renewable energy cost before the Inflation Reduction Act (pre-IRA) compared to after the Inflation Reduction Act (post-IRA). These marginal generator counts are reported in Cambium 2021 for pre-IRA[1] and Cambium 2022 for post-IRA.[18]

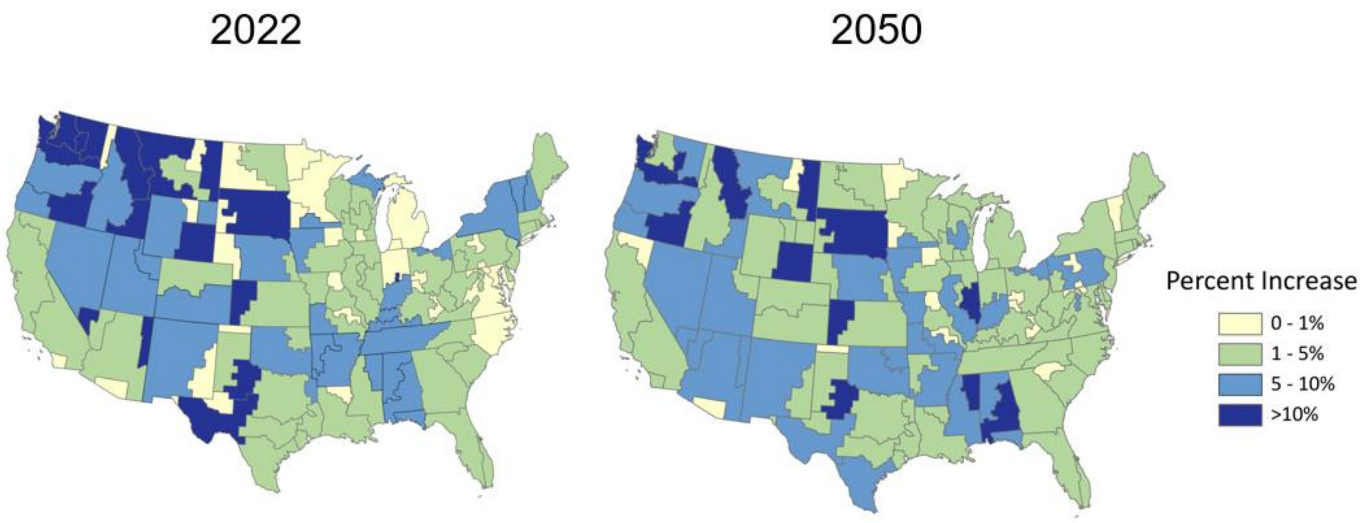

Figure S1. Percentage change in total electricity demand for each balancing area with the electrification of all major trucking corridors.

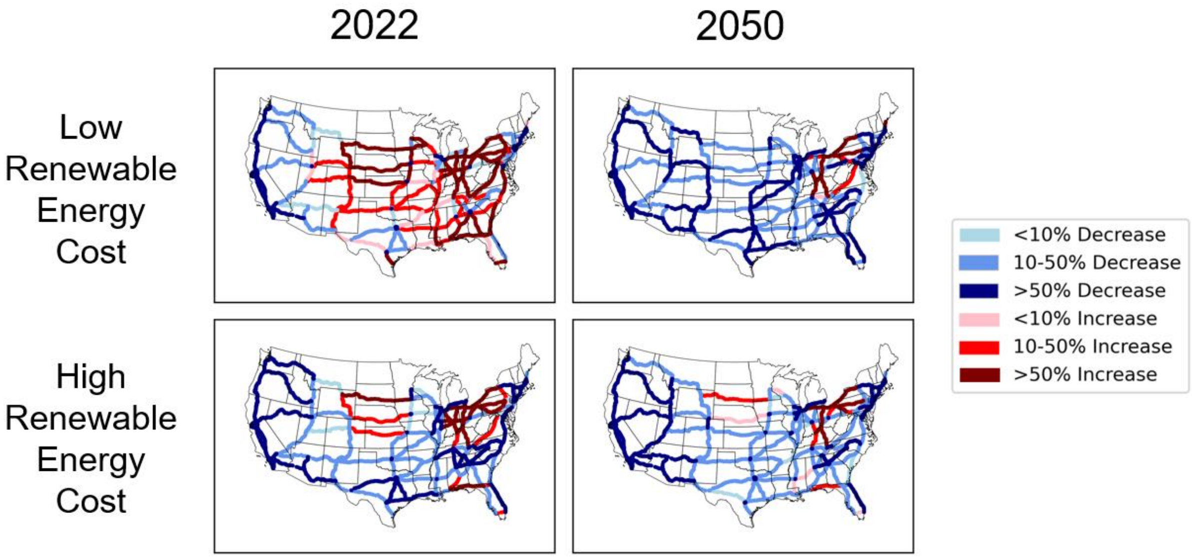

Figure S2. Corridor analysis showing percentage change in air pollution-related mortality over time for the switch to electric trucks compared to the base-case of diesel trucks. Two electricity grid scenarios are shown: (1) low renewable energy cost (upper panel), and (2) high renewable energy cost (lower panel).

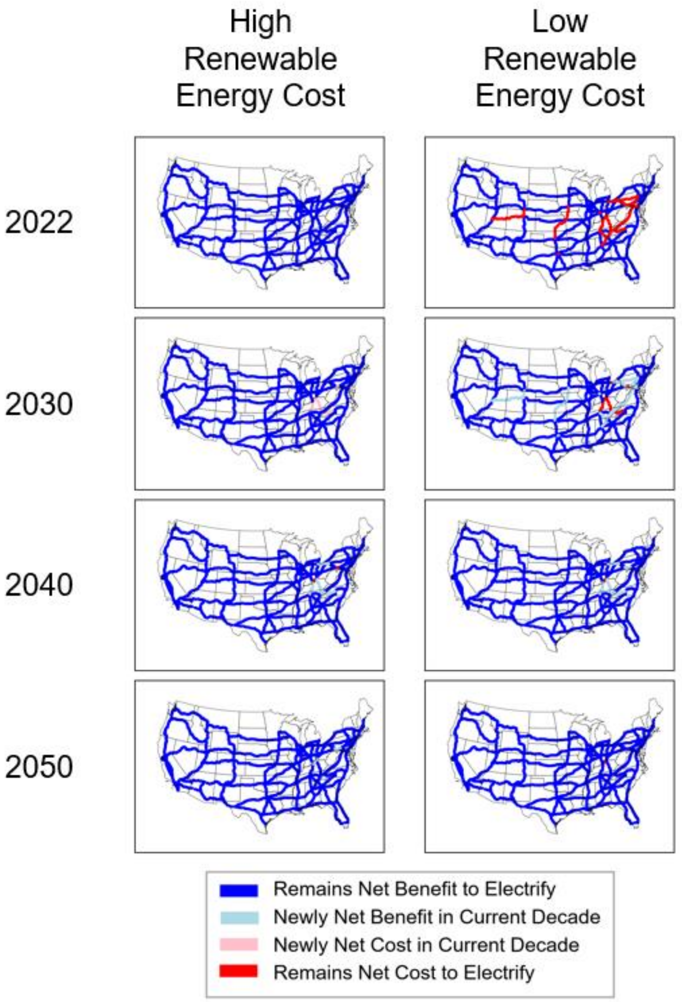

Figure S3. Corridor analysis showing which corridors are beneficial to electrify for reducing greenhouse gas emissions compared to diesel trucks. We show how beneficial corridors change over time as regional electricity grid mixes change. Two electricity grid scenarios are shown: (1) low renewable energy cost (right column), and (2) high renewable energy cost (left column).

Table S1. Comparison of Total Life-Cycle Health and Climate Damages Across All Corridors for Diesel Trucks and Electric Trucks (Unit = Dollars).

	Diesel Trucks	Low Renewable Energy Cost Electric Trucks	Mid-case Renewable Energy Cost Electric Trucks	High Renewable Energy Cost Electric Trucks
**2022**	1.36E+10	1.41E+10	1.45E+10	1.01E+10
**2030**	1.51E+10	1.39E+10	1.42E+10	1.44E+10
**2040**	1.70E+10	1.14E+10	1.43E+10	1.41E+10
**2050**	1.97E+10	1.10E+10	1.21E+10	1.32E+10

<10% Decrease
10-50% Decrease
>50% Decrease
<10% Increase
10-50% Increase
>50% Increase

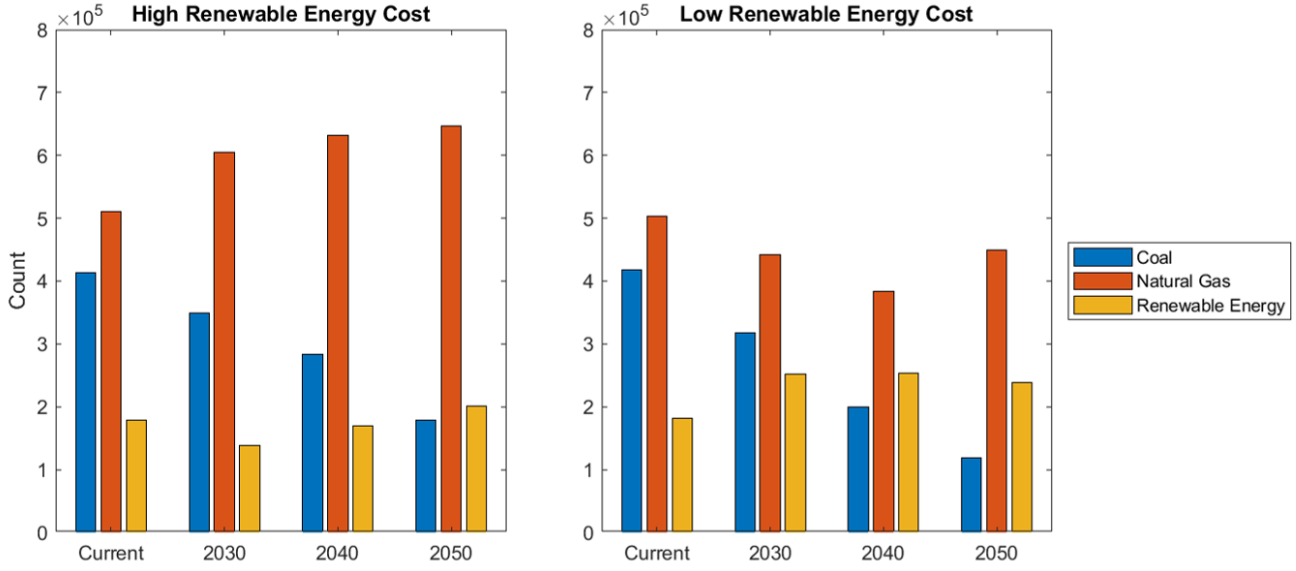

Figure S4. The number of times coal, natural gas, and renewable energy act as the hourly marginal generator for any balancing area in a given year as determined by NREL's Cambium 2021.[1]

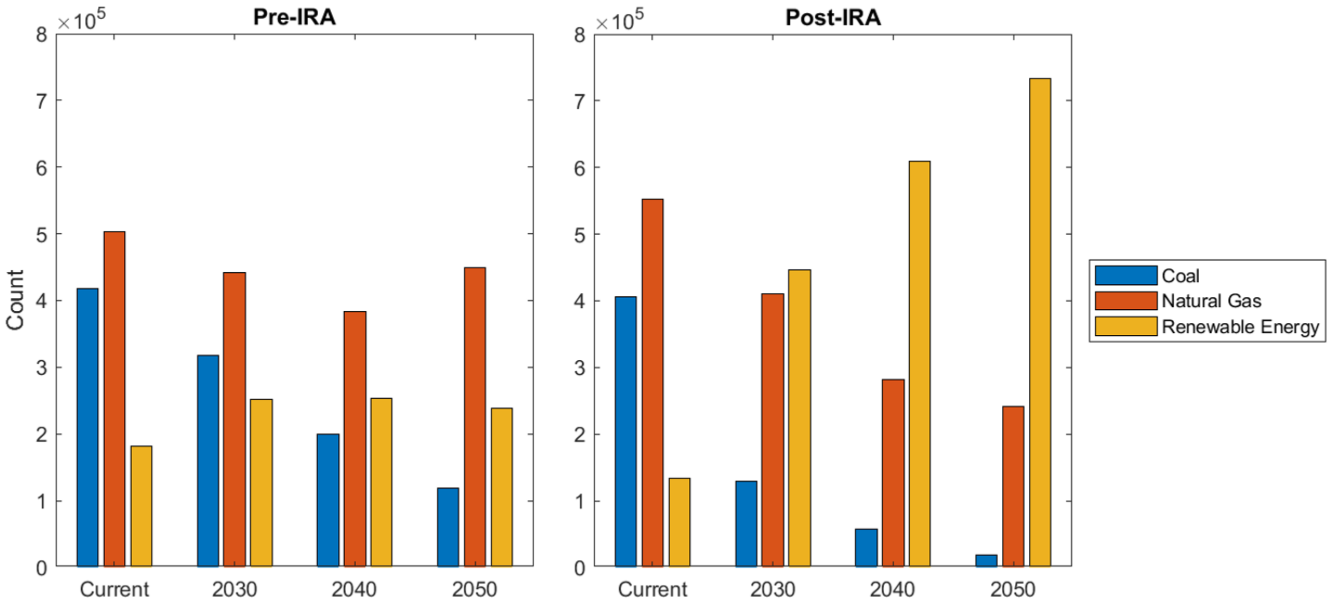

Figure S5. The number of times coal, natural gas, and renewable energy act as the hourly marginal generator for any balancing area in a given year under a low renewable energy cost scenario pre-IRA[1] vs. post-IRA.[18] (IRA is short for Inflation Reduction Act.)

Table S2. Comparison of total air pollution-related premature mortality, measured in total deaths, across all corridors for diesel vs. electric trucks (pre- and post-IRA). Bold entries indicate a net reduction in damages relative to diesel trucks. (IRA is short for Inflation Reduction Act.)

Year	MY 2010 - 2018 Diesel Trucks	Grid Scenario: Renewable Energy Cost (Pre-IRA)			Grid Scenario: Renewable Energy Cost (Post-IRA)		
		High	Mid-Case	Low	High	Mid-Case	Low
Near Future	568	580	825	788	**431**	829	794
2030	630	780	803	780	762	**325**	**317**
2040	710	750	761	**616**	**676**	**352**	**189**
2050	824	**601**	**556**	**507**	**511**	**203**	**119**

Table S3. Fraction of Interstate vs. Drayage Corridors that are Beneficial to Electrify from an Air Pollution-Related Mortality Standpoint.

Corridor Type	Near Future Grid: Renewable Energy Cost			2050 Grid: Renewable Energy Cost		
	High	Mid-Case	Low	High	Mid-Case	Low
Interstate	68%	20%	25%	66%	79%	86%
Drayage	91%	76%	76%	98%	98%	98%

Table S4. Fraction of Vehicle Miles Traveled (VMT) with Net Benefits.

	**Low Renewable Energy Cost Electric Trucks**	**Mid-case Renewable Energy Cost Electric Trucks**	**High Renewable Energy Cost Electric Trucks**
**2022**	44.8%	42.8%	85.9%
**2030**	73.5%	73.2%	60.2%
**2040**	88.4%	80.8%	81.3%
**2050**	94.2%	89.3%	90.0%

Table S5. Fraction of Road Miles with Net Benefits.

	**Low Renewable Energy Cost Electric Trucks**	**Mid-case Renewable Energy Cost Electric Trucks**	**High Renewable Energy Cost Electric Trucks**
**2022**	52.5%	48.0%	90.2%
**2030**	79.8%	77.1%	68.5%
**2040**	92.7%	86.3%	86.9%
**2050**	96.0%	93.4%	93.9%

Table S6. Comparison of total air pollution-related premature mortality, measured in total deaths, across all corridors for base-case battery pack specific energy electric trucks (240 Wh/kg) vs. optimistic battery pack specific energy electric trucks (320 Wh/kg).

	Base-Case Battery Technology			Optimistic Battery Technology		
	Low Renewable Energy Cost Electric Trucks	Mid-case Renewable Energy Cost Electric Trucks	High Renewable Energy Cost Electric Trucks	Low Renewable Energy Cost Electric Trucks	Mid-case Renewable Energy Cost Electric Trucks	High Renewable Energy Cost Electric Trucks
**Current**	788	825	580	784	820	570
**2030**	780	803	780	767	789	772
**2040**	616	761	750	613	756	747
**2050**	507	556	601	499	549	593

Table S7. Comparison of total air pollution-related premature mortality, measured in total deaths, across all corridors for base-case electric and diesel truck design vs. future electric and diesel truck design.

	Base-Case Truck Design				Future Truck Design			
	MY 2010 - 2018 Diesel Trucks	Low Renewable Energy Cost Electric Trucks	Mid-case Renewable Energy Cost Electric Trucks	High Renewable Energy Cost Electric Trucks	MY 2010 - 2018 Diesel Trucks	Low Renewable Energy Cost Electric Trucks	Mid-case Renewable Energy Cost Electric Trucks	High Renewable Energy Cost Electric Trucks
**Current**	568	788	825	580	554	700	732	509
**2030**	630	780	803	780	615	684	704	689
**2040**	710	616	761	750	693	546	674	667
**2050**	824	507	556	601	804	445	490	529

Table S8. List of Scenarios Used from NREL's Standard Scenarios.[19,20]

Acronym	NREL Standard Scenario
High Renewable Energy Cost (Before IRA)	2021 Standard Scenarios: High Renewable Energy Costs
Mid-Case Renewable Energy Cost (Before IRA)	2021 Standard Scenarios: Mid-Case
Low Renewable Energy Cost (Before IRA)	2021 Standard Scenarios: Low Renewable Energy Cost
High Renewable Energy Cost (After IRA)	2022 Standard Scenarios: High Renewable Energy and Battery Costs (phaseout threshold not reached)
Mid-Case Renewable Energy Cost (After IRA)	2022 Standard Scenarios: Mid-case (without tax credit phaseout)
Low Renewable Energy Cost (After IRA)	2022 Standard Scenarios: Low Renewable Energy and Battery Costs (without tax credit phaseout)

Table S9. Normalized air pollution-related premature mortality, measured in deaths per million people, across all corridors.

	MY 2010 - 2018 Diesel Trucks	Low Renewable Energy Cost Electric Trucks	Mid-case Renewable Energy Cost Electric Trucks	High Renewable Energy Cost Electric Trucks
**2022**	1.67	2.32	2.43	1.71
**2030**	1.85	2.30	2.36	2.30
**2040**	2.09	1.81	2.24	2.21
**2050**	2.43	1.49	1.64	1.77

Table S10. Total use-phase air pollution-related mortality from heavy-duty truck corridors (Unit = mortality).

	2022				2030				2040				2050			
Corridor	Diesel	Low RE Cost	Mid-Case	High RE Cost	Diesel	Low RE Cost	Mid-Case	High RE Cost	Diesel	Low RE Cost	Mid-Case	High RE Cost	Diesel	Low RE Cost	Mid-Case	High RE Cost
1	2.9	0.58	0.55	0.64	3.2	0.58	0.87	0.6	3.6	0.94	0.63	0.75	4.2	1.6	1.3	0.9
2	4.8	0.99	0.89	0.79	5.4	0.95	1.1	1.1	6.1	1.3	0.92	0.84	7	2.7	2.3	1
3	3.3	0.44	0.42	0.37	3.6	0.35	0.44	0.35	4.1	0.51	0.39	0.33	4.7	0.83	0.69	0.47
4	3.8	1.7	1.7	1.5	4.2	1.6	1.9	1.6	4.7	1.6	1.7	1.3	5.5	2.6	2.7	1.7
5	6.3	1.4	1.4	1.1	7	1.1	1.3	1.1	7.9	0.73	1.1	0.73	9.1	1.1	1.5	0.89
6	1.5	0.16	0.16	0.12	1.7	0.13	0.13	0.12	1.9	0.096	0.12	0.1	2.2	0.13	0.16	0.11
7	9.2	1.6	1.6	1.3	10	1.5	1.8	1.5	11	1.4	1.6	1.1	13	2.5	2.5	1.5
8	2.3	0.28	0.27	0.24	2.5	0.23	0.28	0.22	2.8	0.33	0.25	0.21	3.3	0.53	0.44	0.3
9	2.2	0.27	0.26	0.23	2.5	0.22	0.27	0.22	2.8	0.31	0.24	0.21	3.2	0.52	0.43	0.29
10	0.44	0.4	0.42	0.22	0.48	0.29	0.45	0.3	0.55	0.27	0.26	0.28	0.63	0.33	0.25	0.31
11	0.47	0.51	0.5	0.29	0.52	0.35	0.48	0.35	0.59	0.21	0.3	0.29	0.68	0.28	0.31	0.32
12	0.64	0.66	0.63	0.4	0.71	0.46	0.6	0.47	0.8	0.29	0.39	0.38	0.93	0.32	0.39	0.4
13	3.2	2.8	2.9	2.3	3.6	2.8	2.9	2.5	4	1.6	2.4	1.6	4.7	2.5	3.2	1.9
14	5	0.65	0.65	0.56	5.5	0.6	0.63	0.58	6.2	0.45	0.59	0.49	7.2	0.59	0.73	0.53
15	0.54	0.45	0.47	0.27	0.6	0.41	0.37	0.34	0.68	0.28	0.27	0.28	0.79	0.43	0.32	0.35
16	0.43	0.67	1.1	0.6	0.48	0.44	0.58	0.55	0.54	0.27	0.42	0.38	0.63	0.36	0.37	0.45
17	1	1.2	1.3	0.63	1.1	0.73	1.1	0.75	1.2	0.49	0.67	0.57	1.4	0.57	0.62	0.72
18	1.6	1.8	2	1.1	1.7	1.6	1.7	1.4	1.9	0.79	1.1	0.98	2.3	0.89	1	1.2
19	0.8	1.1	1.1	0.63	0.89	0.92	0.87	0.89	1	0.47	0.67	0.58	1.2	0.53	0.62	0.68
20	1.8	1.6	1.6	0.89	2	1	1.2	1.4	2.3	0.57	1.3	1.4	2.7	0.79	0.85	1.4
21	2.2	3.6	3.6	2.1	2.5	2.3	2.7	3.2	2.8	1.5	3	3	3.2	1.9	2.1	3.1
22	1.9	2.8	2.8	1.7	2.1	2.2	2.4	2.6	2.3	1.6	2.6	2.5	2.7	1.8	1.8	2.3
23	6.2	7.4	7.4	4.6	6.9	5.7	6.2	6.6	7.7	4	6.2	5.3	9	4.1	4.8	5.5
24	6.4	6.2	6.4	3.3	7.1	6.2	5.5	7.2	8	3.8	6.1	5.1	9.3	4.4	5.1	7.4
25	19	14	15	6.7	21	16	13	16	24	7.5	13	13	28	8.5	10	16
26	3.8	6.1	6.5	2.6	4.2	4.8	5.3	5.4	4.8	2.3	4.1	5.3	5.5	2.7	3.2	4.5
27	1.8	0.94	0.98	0.89	2	0.99	1.6	1.1	2.3	1.4	1	1.3	2.6	2	1.4	1.4
28	1.1	1.1	1.9	1	1.3	0.81	1.1	0.92	1.4	0.55	0.81	0.79	1.7	0.66	0.76	0.92
29	3.3	7.8	9.5	5.7	3.7	4.2	8	6.6	4.2	2.4	5.2	5.7	4.9	2.7	3.6	5.5
30	2.6	1.4	1.4	0.89	2.9	1.1	1.4	1	3.3	1	0.87	0.9	3.8	1.8	1.6	0.86
31	3.1	2.2	2.2	1.5	3.4	2.5	2	1.5	3.9	1.8	1.4	1.5	4.5	2.4	1.9	1.7
32	3.2	4	3.9	2.7	3.6	3.3	5	3.2	4	2	2.5	2.2	4.7	2.1	2.2	2.6
33	14	25	25	17	16	17	21	24	18	12	20	22	20	12	15	21
34	4.8	7.1	8.6	4.3	5.3	5.5	7.8	5	6	3.4	4.5	4.2	6.9	3.8	4.4	5
35	5.3	9.3	9.8	6.3	5.9	6.1	7.7	7.9	6.6	4.4	6.5	6.6	7.7	4.4	5.4	6.8
36	0.82	0.81	0.83	0.57	0.91	0.71	0.8	0.69	1	0.59	0.6	0.59	1.2	0.68	0.84	0.56
37	1.6	1.5	1.6	0.87	1.7	1.4	1.2	1.2	1.9	0.79	0.86	0.77	2.3	0.99	0.88	0.94
38	5.1	6.1	6	4	5.6	5.4	5.5	6.5	6.3	3.9	5.6	5.2	7.4	3.9	4.9	6.2
39	7.2	8.6	8.8	5	7.9	7.6	7.5	8.3	9	5.3	8.1	6.6	10	5.6	6.8	7.7

40	6.5	7.1	7.4	3.4	7.3	8.7	7.1	8.4	8.2	4.7	8	6.6	9.5	5	6.3	8.4
41	6.4	4.4	4.4	2.5	7.1	6.6	6	6.9	8	4.2	6.5	7	9.3	4.6	5	6.6
42	7.7	2.6	2.6	2.1	8.5	2.5	2.6	2.3	9.6	1.5	2.2	1.4	11	2.6	2.9	1.7
43	3	2.1	2.2	1.3	3.3	2	1.8	1.7	3.8	1.3	1.3	1.3	4.4	1.9	1.5	1.6
44	1.6	1.6	1.6	0.93	1.7	1.9	1.8	1.9	2	1	1.6	1.8	2.3	1.2	1.2	1.5
45	4.5	4.6	4.8	2.6	5	6.1	6.4	6.5	5.6	3.6	5.9	7.6	6.5	4.2	4.4	6
46	5.2	4.6	4.6	1.9	5.8	4.5	5	4.8	6.5	2.3	3.9	4.8	7.5	2.5	3	4.1
47	5.2	4.2	4	1.8	5.8	4.9	4.6	5.2	6.5	2.5	4.3	4.3	7.5	2.8	3.4	5.1
48	2.5	4.4	4.5	2.3	2.8	3.3	3.4	3.9	3.2	2	3.9	4	3.7	2.8	2.9	3.7
49	0.91	1.5	1.5	0.82	1	1	1.1	1.4	1.1	0.63	1.2	1.2	1.3	0.83	0.94	1.3
50	12	17	17	12	14	16	16	16	15	11	16	16	18	11	12	15
51	6	6.5	6.6	4.3	6.6	6.3	6.9	6.2	7.5	4.3	6.6	5.9	8.7	4.3	4.7	5.6
52	8.7	9.8	9.8	6.3	9.6	8.9	9.7	9.7	11	6	9.4	8.6	13	6	6.9	8.6
53	5.2	7.4	7.8	3.1	5.8	7.8	7.7	7.1	6.5	4.7	8.2	6.8	7.6	4.8	6.3	6.9
54	7.1	8.6	9.1	4	7.9	10	8.9	9.3	8.9	6	9.4	8	10	6.2	7.2	9.2
55	6.3	6.6	6.1	2.9	7	7.5	7.3	6.8	7.9	4.2	6.5	6.5	9.2	4	4.9	5.9
56	2.8	2.9	2.9	2.1	3.1	3.4	3.2	2.9	3.5	2.2	2.8	2.8	4	1.9	2.2	2.5
57	1.8	2.3	2.4	1.3	2	2.3	2.3	2.1	2.3	1.5	2.2	2.1	2.7	1.4	1.6	1.8
58	2.1	1.6	1.7	1	2.4	1.8	1.8	1.6	2.7	1.1	1.7	1.5	3.1	1.1	1.2	1.4
59	3	3.3	3.5	1.4	3.3	3	3.3	3	3.8	1.9	3.2	2.7	4.4	2	2.2	2.7
60	1.2	1.5	1.5	0.99	1.4	1.6	1.9	1.5	1.6	1.1	1.7	1.5	1.8	1.1	1.2	1.4
61	1.8	2.6	2.7	1.5	2	2.8	3	2.6	2.2	1.8	2.9	2.5	2.6	1.8	2.2	2.5
62	4	5	5.2	2.3	4.5	5.7	5.4	4.9	5.1	3.4	5.6	4.7	5.9	3.7	4.4	5
63	2.1	3.2	3.4	1.4	2.3	3.5	3.4	3.1	2.6	2.2	3.5	3	3	2.3	2.7	3.2
64	1.8	1.7	1.8	1	2	1.9	1.9	1.7	2.2	1.2	1.7	1.6	2.6	1.1	1.3	1.5
65	1.6	1.8	1.9	0.76	1.8	1.5	1.7	1.5	2	0.95	1.7	1.5	2.3	1	1.1	1.3
66	3	4.1	4.3	2.3	3.3	4.1	4.7	3.9	3.7	2.7	4.4	3.9	4.3	2.7	3.1	3.7
67	6.5	12	13	10	7.2	14	14	11	8.1	9.6	11	11	9.4	6.6	7.7	7.8
68	4.1	9.7	9.8	8.9	4.6	11	12	9.2	5.2	8.5	9.4	9.1	6	9.1	8.1	7.7
69	5.7	9.3	9.7	6.6	6.3	10	10	11	7.1	9.2	11	11	8.3	15	11	10
70	3.5	3.5	3.7	1.7	3.9	4	3.8	3.5	4.4	2.7	3.7	3.2	5.1	3.9	2.7	3.5
71	3.1	3.5	3.6	2	3.5	3.5	3.7	3.4	3.9	2.3	3.7	3.4	4.5	2.7	2.8	2.7
72	1.6	3.1	3.1	2.3	1.7	3.1	3.6	3.1	2	2.2	3.6	3.4	2.3	1.8	2.8	2
73	5.5	11	11	9.7	6.1	12	12	9.7	6.9	8.3	9.8	9.4	8	5.4	6.5	5.9
74	4.4	10	11	10	4.9	12	13	11	5.5	12	11	11	6.4	4.6	6.2	5.9
75	6.2	18	18	18	6.9	20	21	19	7.8	20	19	20	9	14	14	14
76	3.4	7.5	7.8	5.4	3.8	9.1	8.7	8.8	4.3	8	9.1	8.9	5	13	9.4	9.1
77	2.1	2.5	2.7	1.2	2.3	2.6	2.4	2.4	2.6	2	2.3	2	3	2.6	1.5	1.7
78	5.9	4.7	4.9	1.7	6.6	4.2	4.1	4.3	7.4	3.1	3.8	3.6	8.6	3.4	2.7	3
79	3.9	6.4	6.6	2.6	4.4	4.6	5.9	6.3	4.9	3.1	5.2	5	5.7	2.8	4	4.3
80	5.7	6	6	5.1	6.3	3.8	6.5	7.5	7.1	3.1	6.6	6.4	8.2	3.8	6.3	6
81	0.22	0.38	0.37	0.25	0.24	0.2	0.33	0.37	0.27	0.16	0.35	0.33	0.32	0.19	0.33	0.32
82	3.1	4.4	4.6	1.4	3.4	4	3.9	3.9	3.9	2.8	3.4	3.1	4.5	3	2.2	2.4
83	1.8	2.3	2.4	0.65	2	2.1	2	2.1	2.2	1.3	1.9	1.7	2.6	1.5	1.3	1.5
84	1.5	2.3	2.4	0.96	1.6	2	2.2	2	1.8	1.3	1.9	1.8	2.1	0.96	1.3	1.2

85	6.3	5.4	5.7	0.92	6.9	5	5.1	4.8	7.8	3.3	4.3	4	9.1	2.4	2.8	3.3
86	1.6	3.5	3.4	2.5	1.8	4.6	4.4	4.4	2	4.2	4.5	4.5	2.3	6.9	5.3	4.7
87	2.8	2.7	2.7	2	3.1	3.3	3.3	3.1	3.5	2.9	3.2	3.2	4	3.9	3.1	3
88	4.1	12	12	12	4.5	13	14	12	5.1	13	12	12	5.9	5.4	7.2	7.6
89	3.4	12	12	11	3.7	14	15	13	4.2	13	13	12	4.9	5.6	7.4	7.8
90	4	6	7.4	5.1	4.4	5.1	3.5	5.9	5	2.5	5.8	6.5	5.7	3.6	4.1	4.1
91	2.1	3.8	4.2	3.7	2.3	3.5	1.7	3.7	2.6	1.3	4	4.3	3.1	2	2.4	2.3
92	12	22	23	13	13	21	17	21	14	14	20	21	17	19	15	14
93	4.2	4.8	5.1	4.2	4.7	4.2	2.9	4.7	5.3	2.5	4.9	5.3	6.2	3.2	3.4	3.3
94	1.6	0.79	1.4	0.33	1.7	0.54	0.56	0.69	2	0.55	0.58	0.64	2.3	0.69	0.58	0.62
95	0.2	0.22	0.82	0.12	0.23	0.17	0.15	0.15	0.26	0.25	0.17	0.16	0.3	0.46	0.3	0.15
96	2.8	1.1	2.7	0.6	3.2	0.93	0.88	1	3.6	1.2	0.91	0.97	4.1	1.9	1.4	0.94
97	10	1.2	1.5	0.77	11	0.99	0.89	1.1	13	0.92	1.1	1.2	15	1.1	0.94	0.91
98	2.8	2.4	2.4	1.7	3.1	2.1	1.8	2.1	3.5	1.9	2.2	2.4	4	2	1.8	1.8
99	3.4	2.6	2.5	1.6	3.8	2.1	2	2.2	4.2	1.9	2.2	2.3	4.9	1.9	1.9	1.7
100	3.3	5.2	5.3	1	3.6	4.5	4.8	4.7	4.1	3.9	4.4	4.4	4.7	4.6	4	3.9
101	1.4	1.8	1.8	0.45	1.5	1.4	1.5	1.6	1.7	1.1	1.4	1.5	2	1.2	1.2	1.3
102	2.3	3.7	3.7	1.9	2.6	2.6	3.5	3.9	2.9	2	3.3	3.3	3.4	2.1	2.8	3.1
103	4.6	3.1	3.1	2	5.1	2	3.3	3.5	5.8	1.5	3.4	3.3	6.7	1.8	3.2	3.2
104	5.1	6.2	6.5	3.9	5.7	6.4	6.2	6.2	6.4	4.2	6.1	6	7.4	4.4	5	5.5
105	6.9	3.2	3.3	2.3	7.7	3.8	3.7	3.3	8.6	2.5	3.2	3.1	10	2.2	2.5	2.9
106	2.7	0.95	0.95	0.76	3	1	0.94	0.89	3.3	0.65	0.84	0.85	3.9	0.53	0.66	0.71
107	3.4	1.6	1.6	1	3.7	1.7	1.5	1.5	4.2	1.2	1.5	1.4	4.9	0.95	1.1	1.2
108	2	1.8	2	0.93	2.2	2.1	1.9	1.9	2.4	1.7	2.1	1.8	2.8	1.2	1.4	1.3
109	1.2	0.64	0.69	0.38	1.3	0.7	0.7	0.66	1.5	0.63	0.7	0.55	1.7	0.36	0.43	0.34
110	2.8	2.3	2.4	1.7	3.1	2.7	2.6	2.4	3.5	1.7	2.3	2.3	4.1	1.6	1.8	2
111	14	49	52	47	15	51	53	48	17	54	50	50	20	24	30	32
112	5.2	42	43	43	5.8	48	53	45	6.5	48	44	44	7.6	18	25	25
113	5.7	32	35	31	6.4	32	34	31	7.2	35	32	32	8.3	15	19	20
114	1.8	1.3	2.4	0.55	2	0.94	0.97	1.2	2.3	0.92	0.99	1.1	2.6	1.2	0.99	1.1
115	1.1	0.57	1.3	0.31	1.2	0.45	0.42	0.49	1.4	0.57	0.45	0.47	1.6	0.95	0.7	0.46
116	2	15	15	14	2.2	16	18	15	2.5	17	15	15	2.9	6	8.4	8.5
117	6.2	34	36	35	6.9	34	34	34	7.7	33	35	36	9	16	21	21
118	2.8	3.1	3.3	2.7	3.1	2.8	1.9	3	3.5	1.6	3.2	3.4	4.1	2.1	2.2	2.1
119	0.61	0.16	0.2	0.096	0.67	0.13	0.12	0.13	0.76	0.12	0.14	0.15	0.88	0.13	0.12	0.12
120	0.68	0.11	0.2	0.04	0.75	0.07	0.072	0.083	0.85	0.071	0.077	0.083	0.98	0.096	0.077	0.079
121	1.1	1.3	1.4	0.55	1.3	1.1	1.2	1.1	1.4	0.66	1.2	1.1	1.6	0.7	0.76	0.88
122	1.5	3.1	3.2	2.6	1.7	3.2	3.5	2.7	1.9	2.2	2.7	2.8	2.2	1.5	1.9	1.8
123	4.7	7.9	8.2	7.5	5.2	8.6	9.3	7	5.8	6	7.1	7	6.7	3.9	4.5	4.7
124	2.9	2.7	3	1.9	3.2	3.2	3	2.7	3.6	2.6	3	3	4.2	1.7	1.8	2.4
125	3.2	9.2	10	7.6	3.6	9.1	6.3	9.3	4	5	9.9	10	4.7	5.3	6	6.7
126	2	4.6	5.1	4.4	2.3	4.1	2.1	4.5	2.6	1.5	4.8	5.2	3	2.4	2.9	2.8
127	5.8	3.4	3.6	3	6.4	2.9	2	3.3	7.2	1.7	3.4	3.7	8.4	2.2	2.4	2.3
128	3.3	4	4.2	3.3	3.6	3.4	2.4	3.7	4.1	1.8	3.9	4.1	4.8	2.2	2.6	2.5
129	0.64	0.35	0.36	0.24	0.71	0.31	0.26	0.31	0.8	0.27	0.33	0.35	0.93	0.29	0.27	0.26

130	0.72	0.4	0.41	0.28	0.8	0.35	0.3	0.35	0.91	0.31	0.37	0.4	1.1	0.33	0.3	0.3
131	0.88	1.2	1.2	0.49	0.97	1	1.1	1	1.1	0.63	1.1	0.92	1.3	0.68	0.72	0.87
132	2.8	6.4	6.8	5.6	3.1	6.8	7.3	5.8	3.5	4.6	5.7	5.8	4.1	3.2	3.9	3.9
133	5.4	5.5	5.9	2.7	6	6.5	6.1	5.6	6.8	4.3	6	5.2	7.9	5.1	4.5	5.1
134	2	2.9	3	1.4	2.3	3.2	2.9	2.8	2.6	2.4	2.8	2.5	3	3.2	1.9	2.2
135	3	3.5	3.8	1.4	3.4	3.8	3.8	3.6	3.8	2.9	3.5	3.3	4.4	3.1	2.5	2.9
136	4.3	3.4	3.5	0.8	4.8	3.1	3.5	3.3	5.4	2.6	3.2	3.2	6.2	2.4	2.5	2.8
137	2.8	3.7	3.9	1.7	3.1	4.2	4	3.7	3.5	2.6	4.1	3.5	4	3.6	3.2	4.1
138	2.6	3	3.1	0.81	2.9	2.7	2.6	2.6	3.3	1.7	2.4	2.2	3.8	1.8	1.6	1.9
139	3.8	5.3	5.4	0.92	4.2	4.4	4.5	4.5	4.7	2.7	3.6	3.5	5.5	2.2	2.5	3
140	2.4	3	3.1	1.8	2.6	3.2	3.4	3	3	2.1	3.4	3.1	3.4	1.9	2.6	2.5
141	3.1	7	6.4	5	3.4	6	7.1	6.6	3.8	4.6	6.9	6.8	4.5	3.4	5.8	4.9
142	0.37	0.57	0.56	0.47	0.41	0.29	0.53	0.59	0.46	0.26	0.54	0.51	0.54	0.32	0.53	0.47
143	0.47	0.21	0.21	0.03	0.53	0.19	0.19	0.17	0.59	0.12	0.15	0.14	0.69	0.085	0.099	0.11
144	0.32	0.17	0.17	0.024	0.35	0.15	0.15	0.14	0.4	0.098	0.12	0.12	0.46	0.069	0.08	0.085
145	0.18	0.1	0.1	0.015	0.2	0.093	0.092	0.084	0.22	0.06	0.075	0.07	0.26	0.042	0.049	0.052
146	0.44	0.22	0.22	0.031	0.49	0.2	0.2	0.18	0.55	0.13	0.16	0.15	0.64	0.09	0.1	0.11
147	0.96	0.33	0.33	0.047	1.1	0.3	0.3	0.27	1.2	0.19	0.24	0.23	1.4	0.14	0.16	0.17
148	1.1	0.4	0.39	0.056	1.3	0.36	0.35	0.32	1.4	0.23	0.29	0.27	1.6	0.16	0.19	0.2
149	0.47	0.18	0.18	0.084	0.52	0.11	0.12	0.13	0.59	0.059	0.099	0.14	0.68	0.061	0.08	0.1
150	1.1	0.4	0.42	0.17	1.3	0.38	0.31	0.44	1.4	0.21	0.36	0.31	1.6	0.26	0.33	0.46
151	0.73	0.37	0.37	0.18	0.82	0.35	0.28	0.39	0.92	0.2	0.33	0.3	1.1	0.22	0.29	0.4
152	0.8	0.42	0.42	0.2	0.88	0.39	0.31	0.44	1	0.22	0.37	0.34	1.2	0.24	0.32	0.45
153	0.81	0.28	0.29	0.13	0.9	0.26	0.21	0.3	1	0.15	0.25	0.23	1.2	0.17	0.22	0.3
154	1.4	0.42	0.43	0.2	1.5	0.39	0.32	0.45	1.7	0.22	0.37	0.34	2	0.25	0.33	0.46
155	3.9	0.14	0.14	0.12	4.4	0.15	0.15	0.13	4.9	0.081	0.12	0.068	5.7	0.15	0.18	0.074
156	1.1	0.81	0.83	0.43	1.2	0.84	0.73	0.74	1.3	0.68	0.72	0.66	1.6	0.91	0.47	0.52
157	1.4	1.7	1.8	0.82	1.6	1.7	1.5	1.5	1.8	1.3	1.5	1.3	2.1	1.8	0.95	1
158	0.91	0.25	0.8	0.17	1	0.22	0.18	0.19	1.1	0.37	0.22	0.2	1.3	0.8	0.47	0.2
159	0.61	0.2	0.64	0.13	0.67	0.17	0.15	0.15	0.76	0.31	0.18	0.16	0.88	0.66	0.38	0.16
160	0.89	0.49	0.45	0.16	0.99	0.36	0.38	0.37	1.1	0.33	0.37	0.37	1.3	0.34	0.33	0.25
161	3.8	14	14	13	4.2	15	17	14	4.7	16	14	14	5.5	5.6	7.9	8
162	1.5	4.2	4.2	4	1.7	4.7	5.1	4.4	1.9	4.8	4.4	4.4	2.2	1.7	2.4	2.4
163	0.78	3.1	3.2	3	0.87	3.5	3.9	3.4	0.98	3.7	3.4	3.3	1.1	1.3	1.8	1.9
164	1.3	0.097	0.092	0.07	1.4	0.073	0.078	0.066	1.6	0.046	0.07	0.045	1.8	0.083	0.099	0.051
165	2.1	0.19	0.18	0.14	2.3	0.14	0.15	0.13	2.6	0.089	0.14	0.086	3	0.16	0.19	0.098
166	0.43	0.021	0.021	0.018	0.48	0.02	0.023	0.019	0.54	0.03	0.022	0.017	0.62	0.052	0.042	0.024
167	0.092	0.0059	0.0057	0.0051	0.1	0.0055	0.0063	0.0052	0.11	0.0083	0.0061	0.0047	0.13	0.014	0.012	0.0066
168	0.053	0.0029	0.0028	0.0025	0.059	0.0027	0.0031	0.0025	0.066	0.004	0.003	0.0023	0.077	0.0071	0.0058	0.0032
169	0.18	0.078	0.079	0.062	0.2	0.082	0.077	0.073	0.22	0.053	0.069	0.07	0.26	0.044	0.054	0.059
170	0.4	0.18	0.18	0.15	0.45	0.19	0.18	0.17	0.51	0.12	0.16	0.16	0.59	0.1	0.13	0.14
171	0.37	0.16	0.16	0.13	0.41	0.17	0.16	0.15	0.46	0.11	0.14	0.14	0.53	0.09	0.11	0.12
172	0.47	0.37	0.37	0.24	0.52	0.3	0.33	0.32	0.59	0.2	0.33	0.29	0.68	0.19	0.23	0.26
173	0.18	0.22	0.24	0.11	0.2	0.18	0.2	0.19	0.22	0.12	0.21	0.19	0.26	0.11	0.13	0.16

174	0.099	0.061	0.062	0.032	0.11	0.063	0.055	0.056	0.12	0.051	0.054	0.049	0.14	0.068	0.035	0.039
175	0.044	0.022	0.022	0.012	0.049	0.023	0.02	0.02	0.055	0.018	0.02	0.018	0.064	0.025	0.013	0.014
176	0.0042	0.0021	0.0022	0.0011	0.0046	0.0022	0.0019	0.0019	0.0052	0.0018	0.0019	0.0017	0.0061	0.0024	0.0012	0.0014
177	0.17	0.097	0.098	0.051	0.19	0.1	0.087	0.089	0.22	0.081	0.085	0.079	0.25	0.11	0.056	0.062
178	1.4	1.5	1.5	1.2	1.5	1.7	1.7	1.9	1.7	1.6	1.8	2	2	2.6	2.1	2
179	0.096	0.055	0.056	0.029	0.11	0.057	0.05	0.051	0.12	0.046	0.049	0.045	0.14	0.062	0.032	0.036
180	1.7	0.58	0.54	0.19	1.9	0.44	0.46	0.45	2.1	0.4	0.44	0.45	2.4	0.4	0.39	0.31
181	1.2	0.7	0.62	0.3	1.3	0.51	0.49	0.5	1.5	0.47	0.5	0.51	1.7	0.37	0.41	0.34
182	0.5	0.29	0.29	0.13	0.56	0.32	0.26	0.28	0.63	0.25	0.3	0.26	0.73	0.19	0.2	0.22
183	0.096	0.034	0.035	0.027	0.11	0.035	0.033	0.03	0.12	0.031	0.031	0.032	0.14	0.016	0.018	0.021
184	0.52	1	1	1	0.57	1	1	0.68	0.65	0.72	0.68	0.67	0.75	0.33	0.44	0.46
185	0.18	0.085	0.087	0.041	0.2	0.084	0.076	0.079	0.23	0.059	0.078	0.076	0.26	0.085	0.058	0.073
186	0.041	0.011	0.011	0.0065	0.046	0.01	0.01	0.009	0.052	0.0052	0.0063	0.0058	0.06	0.0066	0.0073	0.0064
187	1	0.61	0.59	0.42	1.2	0.41	0.53	0.63	1.3	0.33	0.55	0.64	1.5	0.36	0.39	0.52
188	1.1	0.55	0.54	0.38	1.2	0.37	0.48	0.57	1.4	0.3	0.5	0.58	1.6	0.32	0.36	0.47
189	0.042	0.019	0.019	0.013	0.046	0.015	0.018	0.02	0.052	0.012	0.018	0.019	0.06	0.011	0.013	0.017
190	0.088	0.036	0.035	0.022	0.097	0.027	0.03	0.04	0.11	0.023	0.033	0.03	0.13	0.022	0.026	0.035
191	0.56	0.29	0.28	0.18	0.62	0.22	0.24	0.32	0.7	0.19	0.27	0.24	0.81	0.18	0.21	0.28
192	0.57	0.8	0.82	0.79	0.63	0.81	0.81	0.53	0.71	0.57	0.53	0.52	0.82	0.26	0.35	0.36
193	0.86	0.18	0.57	0.12	0.96	0.16	0.13	0.14	1.1	0.27	0.16	0.14	1.3	0.57	0.34	0.14
194	0.067	0.051	0.052	0.024	0.074	0.05	0.046	0.048	0.084	0.035	0.047	0.045	0.097	0.035	0.035	0.039
195	0.15	0.17	0.17	0.082	0.17	0.16	0.15	0.16	0.19	0.1	0.16	0.15	0.22	0.095	0.13	0.14
196	2.5	3.1	3.4	2.9	2.8	2.8	1.4	2.9	3.2	1	3.2	3.5	3.7	1.6	1.8	1.9
197	0.95	0.79	0.8	0.26	1.1	0.76	0.71	0.68	1.2	0.56	0.64	0.59	1.4	0.6	0.41	0.45
198	0.24	0.34	0.35	0.34	0.26	0.34	0.34	0.23	0.3	0.24	0.23	0.22	0.34	0.11	0.15	0.15
199	0.39	0.41	0.44	0.21	0.43	0.34	0.37	0.34	0.48	0.22	0.38	0.34	0.56	0.2	0.24	0.29
200	0.0082	0.0046	0.0046	0.0036	0.0091	0.0039	0.0044	0.0041	0.01	0.0027	0.0042	0.0038	0.012	0.0025	0.003	0.0033

Table S11. Total use-phase greenhouse gas emissions from heavy-duty truck corridors (Unit = tons CO_2 eq.).

	2022				2030				2040				2050			
Corridor	Diesel	Low RE Cost	Mid-Case	High RE Cost	Diesel	Low RE Cost	Mid-Case	High RE Cost	Diesel	Low RE Cost	Mid-Case	High RE Cost	Diesel	Low RE Cost	Mid-Case	High RE Cost
1	7.2E+05	2.6E+05	2.6E+05	2.4E+05	7.9E+05	2.9E+05	3.7E+05	3.2E+05	9.0E+05	2.9E+05	3.9E+05	3.9E+05	1.0E+06	3.1E+05	4.6E+05	5.0E+05
2	2.0E+06	4.8E+05	4.6E+05	4.3E+05	2.2E+06	4.9E+05	6.0E+05	5.6E+05	2.5E+06	5.4E+05	6.2E+05	6.1E+05	2.9E+06	6.1E+05	7.6E+05	6.7E+05
3	4.3E+05	1.5E+05	1.5E+05	1.3E+05	4.8E+05	1.4E+05	1.5E+05	1.5E+05	5.4E+05	1.5E+05	1.8E+05	1.6E+05	6.3E+05	1.4E+05	2.0E+05	1.9E+05
4	1.5E+06	5.6E+05	5.5E+05	5.1E+05	1.7E+06	5.5E+05	6.0E+05	5.7E+05	1.9E+06	4.8E+05	6.7E+05	5.5E+05	2.2E+06	4.5E+05	7.4E+05	6.9E+05
5	5.9E+05	3.6E+05	3.7E+05	3.2E+05	6.5E+05	3.3E+05	3.5E+05	3.3E+05	7.3E+05	2.4E+05	3.9E+05	2.8E+05	8.5E+05	2.2E+05	4.3E+05	3.9E+05
6	1.1E+05	4.5E+04	4.4E+04	3.9E+04	1.2E+05	4.0E+04	4.0E+04	4.1E+04	1.4E+05	3.4E+04	4.8E+04	3.8E+04	1.6E+05	3.2E+04	5.3E+04	4.9E+04
7	1.5E+06	5.0E+05	5.0E+05	4.5E+05	1.6E+06	5.2E+05	5.7E+05	5.3E+05	1.8E+06	4.3E+05	6.1E+05	4.9E+05	2.1E+06	4.2E+05	6.7E+05	6.0E+05
8	2.8E+05	9.5E+04	9.4E+04	8.6E+04	3.1E+05	9.1E+04	9.9E+04	9.7E+04	3.5E+05	9.6E+04	1.2E+05	1.0E+05	4.1E+05	9.1E+04	1.3E+05	1.2E+05
9	2.6E+05	9.1E+04	9.0E+04	8.2E+04	2.9E+05	8.7E+04	9.5E+04	9.4E+04	3.3E+05	9.2E+04	1.1E+05	9.9E+04	3.8E+05	8.8E+04	1.2E+05	1.2E+05
10	3.7E+05	9.1E+04	9.0E+04	5.7E+04	4.1E+05	7.1E+04	9.3E+04	8.4E+04	4.6E+05	4.6E+04	7.6E+04	7.6E+04	5.3E+05	5.2E+04	6.4E+04	8.6E+04
11	2.3E+05	1.4E+05	1.4E+05	9.8E+04	2.6E+05	1.1E+05	1.3E+05	1.3E+05	2.9E+05	8.5E+04	1.3E+05	1.1E+05	3.4E+05	9.6E+04	1.3E+05	1.3E+05
12	2.6E+05	2.0E+05	2.0E+05	1.6E+05	2.9E+05	1.7E+05	1.9E+05	1.9E+05	3.2E+05	1.4E+05	2.1E+05	1.8E+05	3.7E+05	1.6E+05	2.1E+05	2.1E+05
13	1.5E+06	8.3E+05	8.4E+05	7.3E+05	1.7E+06	8.7E+05	8.7E+05	8.5E+05	1.9E+06	5.9E+05	9.2E+05	6.7E+05	2.2E+06	6.0E+05	1.0E+06	8.6E+05
14	4.8E+05	2.0E+05	2.0E+05	1.8E+05	5.4E+05	1.9E+05	2.0E+05	2.0E+05	6.0E+05	1.6E+05	2.3E+05	2.0E+05	7.0E+05	1.5E+05	2.5E+05	2.3E+05
15	1.9E+05	1.7E+05	1.7E+05	1.4E+05	2.1E+05	1.6E+05	1.7E+05	1.7E+05	2.3E+05	9.3E+04	1.7E+05	1.1E+05	2.7E+05	8.4E+04	1.3E+05	1.5E+05
16	2.6E+05	1.6E+05	2.3E+05	1.6E+05	2.8E+05	1.3E+05	1.3E+05	1.7E+05	3.2E+05	9.3E+04	1.4E+05	1.3E+05	3.7E+05	9.8E+04	1.2E+05	1.5E+05
17	2.5E+05	2.8E+05	2.7E+05	1.7E+05	2.7E+05	2.2E+05	2.1E+05	2.4E+05	3.1E+05	1.7E+05	2.3E+05	2.0E+05	3.6E+05	1.9E+05	2.3E+05	2.5E+05
18	5.6E+05	4.9E+05	4.9E+05	3.4E+05	6.2E+05	4.8E+05	4.7E+05	5.0E+05	7.0E+05	3.6E+05	4.7E+05	4.3E+05	8.2E+05	4.0E+05	4.8E+05	4.9E+05
19	3.7E+05	3.2E+05	3.2E+05	2.5E+05	4.1E+05	3.0E+05	3.2E+05	3.4E+05	4.6E+05	2.4E+05	3.1E+05	2.9E+05	5.3E+05	2.6E+05	3.2E+05	3.2E+05
20	3.6E+05	3.3E+05	3.3E+05	2.0E+05	4.0E+05	2.1E+05	2.5E+05	3.2E+05	4.5E+05	1.3E+05	2.8E+05	3.0E+05	5.2E+05	1.6E+05	2.1E+05	3.2E+05
21	6.7E+05	6.6E+05	6.5E+05	4.0E+05	7.5E+05	4.3E+05	4.9E+05	6.2E+05	8.4E+05	2.8E+05	5.6E+05	5.8E+05	9.8E+05	3.6E+05	4.3E+05	6.5E+05
22	5.1E+05	5.3E+05	5.2E+05	3.2E+05	5.6E+05	4.0E+05	4.4E+05	5.2E+05	6.3E+05	2.8E+05	4.8E+05	4.9E+05	7.3E+05	3.3E+05	3.7E+05	5.0E+05
23	1.4E+06	1.4E+06	1.4E+06	9.0E+05	1.5E+06	1.1E+06	1.2E+06	1.4E+06	1.7E+06	7.9E+05	1.2E+06	1.1E+06	2.0E+06	8.0E+05	1.0E+06	1.2E+06
24	1.3E+06	1.2E+06	1.2E+06	6.5E+05	1.4E+06	1.0E+06	9.6E+05	1.2E+06	1.6E+06	6.6E+05	1.0E+06	1.0E+06	1.8E+06	7.4E+05	8.5E+05	1.2E+06
25	3.0E+06	2.7E+06	2.6E+06	1.3E+06	3.3E+06	2.8E+06	2.7E+06	3.2E+06	3.7E+06	1.5E+06	2.4E+06	2.7E+06	4.3E+06	1.7E+06	1.7E+06	2.9E+06
26	1.2E+06	1.1E+06	1.1E+06	5.3E+05	1.3E+06	1.1E+06	1.2E+06	1.3E+06	1.5E+06	6.0E+05	9.7E+05	1.1E+06	1.8E+06	7.1E+05	7.5E+05	1.2E+06
27	1.4E+06	2.5E+05	2.9E+05	2.1E+05	1.6E+06	3.1E+05	4.4E+05	3.7E+05	1.8E+06	2.8E+05	4.3E+05	4.3E+05	2.0E+06	3.4E+05	4.9E+05	5.6E+05
28	8.9E+05	2.9E+05	4.3E+05	2.9E+05	9.8E+05	2.5E+05	2.9E+05	3.1E+05	1.1E+06	1.6E+05	2.8E+05	2.7E+05	1.3E+06	1.7E+05	2.6E+05	3.4E+05
29	1.8E+06	1.4E+06	1.7E+06	1.1E+06	2.0E+06	8.4E+05	1.3E+06	1.3E+06	2.2E+06	4.9E+05	1.1E+06	1.2E+06	2.6E+06	5.4E+05	7.8E+05	1.3E+06
30	1.7E+06	3.6E+05	3.6E+05	2.8E+05	1.9E+06	3.7E+05	4.1E+05	3.8E+05	2.1E+06	2.7E+05	3.3E+05	3.6E+05	2.5E+06	3.2E+05	3.8E+05	3.4E+05
31	1.6E+06	7.5E+05	7.6E+05	6.3E+05	1.8E+06	8.2E+05	6.8E+05	5.7E+05	2.0E+06	6.0E+05	8.5E+05	6.7E+05	2.3E+06	8.9E+05	1.0E+06	9.3E+05
32	1.8E+06	1.2E+06	1.2E+06	9.8E+05	2.0E+06	1.1E+06	1.2E+06	1.2E+06	2.2E+06	7.8E+05	1.1E+06	8.8E+05	2.6E+06	7.8E+05	9.9E+05	1.1E+06
33	5.1E+06	5.1E+06	5.0E+06	3.7E+06	5.7E+06	3.7E+06	4.3E+06	5.3E+06	6.4E+06	2.5E+06	4.4E+06	4.8E+06	7.4E+06	2.7E+06	3.4E+06	4.9E+06
34	1.8E+06	1.9E+06	1.8E+06	1.1E+06	2.0E+06	1.7E+06	1.7E+06	1.7E+06	2.3E+06	1.3E+06	1.8E+06	1.6E+06	2.7E+06	1.5E+06	1.9E+06	1.9E+06
35	1.8E+06	1.8E+06	1.8E+06	1.2E+06	2.0E+06	1.4E+06	1.5E+06	1.8E+06	2.3E+06	1.0E+06	1.5E+06	1.6E+06	2.7E+06	1.0E+06	1.3E+06	1.7E+06
36	5.2E+05	2.8E+05	2.8E+05	2.4E+05	5.8E+05	2.8E+05	3.0E+05	2.9E+05	6.5E+05	1.9E+05	3.2E+05	2.3E+05	7.5E+05	1.9E+05	2.9E+05	2.8E+05
37	7.5E+05	5.0E+05	5.1E+05	3.9E+05	8.4E+05	5.1E+05	5.0E+05	5.0E+05	9.4E+05	3.5E+05	4.4E+05	3.6E+05	1.1E+06	3.6E+05	4.2E+05	4.4E+05
38	1.8E+06	1.4E+06	1.3E+06	1.0E+06	2.0E+06	1.2E+06	1.3E+06	1.6E+06	2.3E+06	9.5E+05	1.4E+06	1.3E+06	2.7E+06	9.8E+05	1.3E+06	1.6E+06
39	1.7E+06	1.6E+06	1.6E+06	9.6E+05	1.9E+06	1.3E+06	1.3E+06	1.6E+06	2.2E+06	9.9E+05	1.5E+06	1.3E+06	2.5E+06	1.1E+06	1.4E+06	1.6E+06
40	1.5E+06	1.3E+06	1.3E+06	6.5E+05	1.7E+06	1.4E+06	1.2E+06	1.5E+06	1.9E+06	8.0E+05	1.3E+06	1.3E+06	2.2E+06	8.7E+05	1.1E+06	1.4E+06
41	1.8E+06	9.0E+05	8.8E+05	5.6E+05	2.0E+06	1.2E+06	1.3E+06	1.4E+06	2.2E+06	8.4E+05	1.3E+06	1.5E+06	2.6E+06	9.6E+05	1.0E+06	1.4E+06
42	1.5E+06	8.1E+05	8.2E+05	7.1E+05	1.7E+06	8.3E+05	8.4E+05	8.2E+05	1.9E+06	5.0E+05	8.8E+05	5.8E+05	2.2E+06	4.8E+05	8.5E+05	7.3E+05
43	1.1E+06	7.6E+05	7.7E+05	6.2E+05	1.2E+06	7.5E+05	7.9E+05	8.0E+05	1.4E+06	4.7E+05	7.9E+05	5.5E+05	1.6E+06	4.6E+05	6.6E+05	7.0E+05
44	6.7E+05	4.0E+05	4.0E+05	2.9E+05	7.4E+05	4.9E+05	5.0E+05	5.4E+05	8.3E+05	3.4E+05	4.8E+05	5.1E+05	9.7E+05	3.9E+05	4.2E+05	4.8E+05
45	1.7E+06	9.5E+05	9.4E+05	5.8E+05	1.9E+06	1.4E+06	1.6E+06	1.6E+06	2.1E+06	8.7E+05	1.4E+06	1.6E+06	2.5E+06	1.0E+06	1.0E+06	1.5E+06
46	9.7E+05	8.3E+05	8.2E+05	3.9E+05	1.1E+06	8.3E+05	9.0E+05	9.5E+05	1.2E+06	4.6E+05	7.3E+05	8.5E+05	1.4E+06	5.2E+05	5.9E+05	8.6E+05
47	8.5E+05	7.5E+05	7.2E+05	3.5E+05	9.5E+05	7.3E+05	7.1E+05	8.2E+05	1.1E+06	4.0E+05	6.4E+05	7.7E+05	1.2E+06	4.6E+05	5.3E+05	7.8E+05
48	9.9E+05	9.5E+05	9.5E+05	5.6E+05	1.1E+06	7.2E+05	8.3E+05	1.0E+06	1.2E+06	4.6E+05	8.8E+05	9.4E+05	1.4E+06	5.5E+05	7.6E+05	1.0E+06
49	3.2E+05	3.0E+05	2.9E+05	1.7E+05	3.5E+05	2.1E+05	2.4E+05	2.9E+05	4.0E+05	1.3E+05	2.6E+05	2.7E+05	4.6E+05	1.6E+05	2.3E+05	3.1E+05
50	3.0E+06	2.8E+06	2.8E+06	1.9E+06	3.3E+06	2.4E+06	2.5E+06	2.8E+06	3.7E+06	1.7E+06	2.6E+06	2.8E+06	4.3E+06	2.0E+06	2.2E+06	2.9E+06
51	1.1E+06	1.1E+06	1.1E+06	7.1E+05	1.3E+06	1.0E+06	1.1E+06	1.1E+06	1.4E+06	7.0E+05	1.1E+06	1.1E+06	1.7E+06	7.3E+05	8.6E+05	1.1E+06
52	1.8E+06	1.7E+06	1.6E+06	1.0E+06	2.0E+06	1.5E+06	1.6E+06	1.8E+06	2.3E+06	1.0E+06	1.6E+06	1.6E+06	2.7E+06	1.0E+06	1.3E+06	1.7E+06
53	1.5E+06	1.4E+06	1.4E+06	6.2E+05	1.7E+06	1.3E+06	1.3E+06	1.4E+06	1.9E+06	8.9E+05	1.5E+06	1.3E+06	2.2E+06	9.8E+05	1.4E+06	1.4E+06

54	1.7E+06	1.6E+06	1.6E+06	7.7E+05	1.9E+06	1.7E+06	1.5E+06	1.7E+06	2.1E+06	1.2E+06	1.7E+06	1.6E+06	2.5E+06	1.3E+06	1.5E+06	1.7E+06
55	1.4E+06	1.1E+06	1.1E+06	5.4E+05	1.6E+06	1.2E+06	1.2E+06	1.2E+06	1.8E+06	7.4E+05	1.1E+06	1.2E+06	2.1E+06	8.6E+05	9.9E+05	1.2E+06
56	4.6E+05	4.0E+05	4.1E+05	2.8E+05	5.1E+05	4.2E+05	4.0E+05	4.1E+05	5.8E+05	2.8E+05	4.0E+05	4.2E+05	6.7E+05	3.1E+05	3.7E+05	4.4E+05
57	4.1E+05	3.7E+05	3.6E+05	2.0E+05	4.5E+05	3.5E+05	3.6E+05	3.6E+05	5.1E+05	2.3E+05	3.6E+05	3.7E+05	5.9E+05	2.6E+05	3.0E+05	3.8E+05
58	3.3E+05	2.7E+05	2.7E+05	1.6E+05	3.6E+05	2.6E+05	2.8E+05	2.8E+05	4.1E+05	1.8E+05	2.8E+05	2.7E+05	4.7E+05	2.1E+05	2.4E+05	3.0E+05
59	7.3E+05	6.2E+05	6.2E+05	2.8E+05	8.1E+05	5.8E+05	6.3E+05	6.3E+05	9.1E+05	4.0E+05	6.6E+05	5.9E+05	1.1E+06	4.6E+05	5.2E+05	6.9E+05
60	2.5E+05	2.3E+05	2.3E+05	1.4E+05	2.7E+05	2.3E+05	2.4E+05	2.4E+05	3.1E+05	1.6E+05	2.5E+05	2.4E+05	3.6E+05	1.7E+05	2.0E+05	2.5E+05
61	5.1E+05	4.4E+05	4.4E+05	2.4E+05	5.6E+05	4.4E+05	4.5E+05	4.5E+05	6.3E+05	3.0E+05	4.9E+05	4.4E+05	7.4E+05	3.2E+05	4.1E+05	4.7E+05
62	1.0E+06	9.5E+05	9.3E+05	4.5E+05	1.2E+06	1.0E+06	9.3E+05	9.8E+05	1.3E+06	6.8E+05	1.1E+06	9.5E+05	1.5E+06	7.5E+05	9.5E+05	1.0E+06
63	6.7E+05	6.0E+05	6.0E+05	2.8E+05	7.4E+05	6.3E+05	5.9E+05	6.1E+05	8.4E+05	4.7E+05	6.8E+05	5.9E+05	9.7E+05	5.4E+05	6.1E+05	6.5E+05
64	3.4E+05	2.9E+05	2.8E+05	1.6E+05	3.8E+05	2.8E+05	2.9E+05	2.9E+05	4.3E+05	1.9E+05	2.9E+05	2.9E+05	4.9E+05	2.2E+05	2.5E+05	3.1E+05
65	3.7E+05	3.4E+05	3.2E+05	1.5E+05	4.1E+05	2.9E+05	3.3E+05	3.3E+05	4.6E+05	2.0E+05	3.4E+05	3.2E+05	5.4E+05	2.3E+05	2.6E+05	3.4E+05
66	8.2E+05	7.1E+05	7.0E+05	3.8E+05	9.1E+05	6.9E+05	7.3E+05	7.2E+05	1.0E+06	4.7E+05	7.5E+05	7.2E+05	1.2E+06	5.1E+05	6.0E+05	7.7E+05
67	1.1E+06	9.6E+05	9.7E+05	6.1E+05	1.2E+06	9.9E+05	9.5E+05	9.6E+05	1.4E+06	7.5E+05	9.3E+05	9.6E+05	1.6E+06	7.5E+05	8.2E+05	9.1E+05
68	7.1E+05	6.9E+05	6.9E+05	5.2E+05	7.9E+05	7.7E+05	7.5E+05	7.1E+05	8.9E+05	6.8E+05	7.1E+05	7.2E+05	1.0E+06	7.5E+05	7.2E+05	7.1E+05
69	1.2E+06	1.3E+06	1.3E+06	8.4E+05	1.4E+06	1.5E+06	1.4E+06	1.4E+06	1.5E+06	1.4E+06	1.4E+06	1.4E+06	1.8E+06	1.6E+06	1.5E+06	1.4E+06
70	7.1E+05	6.6E+05	6.6E+05	3.4E+05	7.9E+05	7.2E+05	6.7E+05	6.9E+05	8.9E+05	5.6E+05	7.3E+05	6.4E+05	1.0E+06	6.0E+05	6.5E+05	7.1E+05
71	6.8E+05	6.2E+05	6.1E+05	3.5E+05	7.6E+05	6.1E+05	5.9E+05	6.2E+05	8.5E+05	4.8E+05	6.7E+05	6.2E+05	9.9E+05	5.4E+05	6.0E+05	5.7E+05
72	6.5E+05	5.9E+05	5.4E+05	4.0E+05	7.2E+05	5.5E+05	5.7E+05	5.8E+05	8.1E+05	4.6E+05	6.3E+05	6.1E+05	9.4E+05	5.0E+05	5.7E+05	4.5E+05
73	9.9E+05	8.4E+05	8.4E+05	6.2E+05	1.1E+06	8.7E+05	8.4E+05	8.4E+05	1.2E+06	6.7E+05	8.5E+05	8.4E+05	1.4E+06	7.0E+05	7.3E+05	7.4E+05
74	5.7E+05	5.1E+05	5.2E+05	3.8E+05	6.4E+05	5.3E+05	5.1E+05	5.2E+05	7.2E+05	4.6E+05	5.3E+05	5.2E+05	8.3E+05	4.2E+05	4.2E+05	4.4E+05
75	9.1E+05	8.9E+05	9.0E+05	6.8E+05	1.0E+06	1.0E+06	9.7E+05	9.5E+05	1.1E+06	9.7E+05	1.0E+06	1.0E+06	1.3E+06	1.0E+06	9.8E+05	9.7E+05
76	9.9E+05	1.1E+06	1.1E+06	7.1E+05	1.1E+06	1.3E+06	1.2E+06	1.2E+06	1.2E+06	1.2E+06	1.2E+06	1.2E+06	1.4E+06	1.3E+06	1.3E+06	1.3E+06
77	4.3E+05	4.4E+05	4.5E+05	2.3E+05	4.8E+05	4.7E+05	4.4E+05	4.6E+05	5.4E+05	4.0E+05	4.5E+05	4.1E+05	6.3E+05	3.9E+05	4.0E+05	4.2E+05
78	9.0E+05	8.6E+05	8.8E+05	3.4E+05	1.0E+06	8.8E+05	8.6E+05	9.2E+05	1.1E+06	7.2E+05	9.0E+05	8.6E+05	1.3E+06	7.0E+05	7.8E+05	8.4E+05
79	1.4E+06	1.2E+06	1.2E+06	6.4E+05	1.5E+06	9.5E+05	1.2E+06	1.3E+06	1.7E+06	8.6E+05	1.3E+06	1.3E+06	2.0E+06	9.6E+05	1.1E+06	1.2E+06
80	1.8E+06	1.5E+06	1.5E+06	1.3E+06	2.0E+06	1.1E+06	1.6E+06	1.7E+06	2.2E+06	1.0E+06	1.7E+06	1.7E+06	2.6E+06	1.3E+06	1.6E+06	1.6E+06
81	1.2E+05	9.8E+04	9.7E+04	5.8E+04	1.3E+05	5.6E+04	8.9E+04	9.9E+04	1.5E+05	6.6E+04	1.0E+05	1.0E+05	1.7E+05	8.3E+04	1.0E+05	1.0E+05
82	6.9E+05	7.1E+05	7.3E+05	2.8E+05	7.6E+05	7.3E+05	7.1E+05	7.6E+05	8.6E+05	6.0E+05	7.4E+05	7.1E+05	1.0E+06	5.7E+05	6.2E+05	6.8E+05
83	3.7E+05	3.8E+05	3.8E+05	1.3E+05	4.1E+05	3.7E+05	3.5E+05	3.9E+05	4.6E+05	2.9E+05	4.0E+05	3.8E+05	5.3E+05	3.0E+05	3.4E+05	3.7E+05
84	3.9E+05	3.6E+05	3.6E+05	1.7E+05	4.3E+05	3.5E+05	3.5E+05	3.7E+05	4.9E+05	2.8E+05	3.7E+05	3.8E+05	5.7E+05	2.8E+05	3.2E+05	3.3E+05
85	9.9E+05	9.4E+05	9.5E+05	2.4E+05	1.1E+06	9.4E+05	9.4E+05	1.0E+06	1.2E+06	7.8E+05	9.8E+05	1.0E+06	1.4E+06	7.5E+05	8.4E+05	9.8E+05
86	3.5E+05	4.2E+05	4.2E+05	2.8E+05	3.9E+05	5.1E+05	4.8E+05	4.7E+05	4.4E+05	5.1E+05	4.8E+05	4.8E+05	5.1E+05	5.9E+05	5.3E+05	4.9E+05
87	4.1E+05	3.7E+05	3.7E+05	2.5E+05	4.5E+05	4.1E+05	4.0E+05	3.9E+05	5.1E+05	3.9E+05	4.0E+05	4.0E+05	5.9E+05	4.3E+05	4.0E+05	4.1E+05
88	6.1E+05	5.8E+05	5.8E+05	4.4E+05	6.8E+05	5.9E+05	5.9E+05	5.8E+05	7.6E+05	5.2E+05	6.0E+05	6.0E+05	8.8E+05	4.8E+05	4.9E+05	5.6E+05
89	5.6E+05	6.0E+05	6.0E+05	4.0E+05	6.2E+05	6.1E+05	6.0E+05	6.0E+05	7.0E+05	5.4E+05	6.2E+05	6.1E+05	8.1E+05	4.9E+05	5.1E+05	5.8E+05
90	8.2E+05	6.9E+05	8.6E+05	4.4E+05	9.2E+05	6.4E+05	5.8E+05	6.8E+05	1.0E+06	5.4E+05	7.0E+05	7.1E+05	1.2E+06	5.7E+05	6.4E+05	6.9E+05
91	3.2E+05	3.5E+05	3.4E+05	2.4E+05	3.6E+05	3.1E+05	2.6E+05	3.2E+05	4.0E+05	2.6E+05	3.4E+05	3.4E+05	4.7E+05	3.1E+05	3.0E+05	3.1E+05
92	3.1E+06	3.3E+06	3.3E+06	1.6E+06	3.5E+06	3.4E+06	3.1E+06	3.4E+06	3.9E+06	2.8E+06	3.4E+06	3.3E+06	4.6E+06	2.9E+06	2.9E+06	3.1E+06
93	5.7E+05	5.5E+05	5.6E+05	3.9E+05	6.3E+05	5.0E+05	4.3E+05	5.2E+05	7.2E+05	4.4E+05	5.6E+05	5.6E+05	8.3E+05	4.8E+05	5.1E+05	5.0E+05
94	3.3E+05	1.7E+05	2.7E+05	8.1E+04	3.6E+05	1.4E+05	1.4E+05	1.7E+05	4.1E+05	1.3E+05	1.6E+05	1.7E+05	4.7E+05	1.2E+05	1.6E+05	1.8E+05
95	1.7E+05	9.3E+04	1.5E+05	6.4E+04	1.9E+05	7.3E+04	7.7E+04	8.7E+04	2.2E+05	6.9E+04	8.8E+04	9.4E+04	2.5E+05	6.9E+04	9.3E+04	1.1E+05
96	5.8E+05	3.0E+05	5.0E+05	1.5E+05	6.4E+05	2.5E+05	2.5E+05	2.9E+05	7.2E+05	2.3E+05	2.9E+05	3.1E+05	8.4E+05	2.2E+05	2.8E+05	3.3E+05
97	3.2E+05	2.1E+05	2.6E+05	1.2E+05	3.6E+05	1.8E+05	1.6E+05	2.0E+05	4.0E+05	1.6E+05	2.0E+05	2.1E+05	4.7E+05	1.6E+05	1.9E+05	2.0E+05
98	4.0E+05	3.5E+05	3.6E+05	2.4E+05	4.4E+05	3.2E+05	2.7E+05	3.3E+05	4.9E+05	2.9E+05	3.5E+05	3.7E+05	5.7E+05	3.0E+05	3.2E+05	3.2E+05
99	4.7E+05	4.2E+05	4.4E+05	2.9E+05	5.2E+05	4.0E+05	3.7E+05	4.1E+05	5.9E+05	3.4E+05	4.4E+05	4.4E+05	6.8E+05	3.6E+05	4.0E+05	3.9E+05
100	1.1E+06	1.0E+06	9.9E+05	2.7E+05	1.2E+06	9.7E+05	9.8E+05	1.0E+06	1.3E+06	8.4E+05	1.0E+06	1.0E+06	1.6E+06	8.9E+05	9.4E+05	1.0E+06
101	4.4E+05	3.9E+05	3.8E+05	1.1E+05	4.9E+05	3.6E+05	3.8E+05	4.0E+05	5.5E+05	3.2E+05	3.9E+05	4.0E+05	6.4E+05	3.5E+05	3.7E+05	4.0E+05
102	1.1E+06	9.1E+05	9.1E+05	4.9E+05	1.2E+06	7.3E+05	8.9E+05	9.9E+05	1.3E+06	6.7E+05	9.4E+05	9.6E+05	1.6E+06	8.0E+05	9.1E+05	9.6E+05
103	1.1E+06	9.0E+05	9.0E+05	5.3E+05	1.3E+06	6.1E+05	9.3E+05	9.7E+05	1.4E+06	5.8E+05	1.0E+06	1.0E+06	1.6E+06	7.7E+05	9.9E+05	1.0E+06
104	1.2E+06	1.1E+06	1.1E+06	6.6E+05	1.3E+06	9.9E+05	1.0E+06	1.1E+06	1.5E+06	6.9E+05	1.1E+06	1.1E+06	1.7E+06	7.8E+05	1.0E+06	1.2E+06
105	5.8E+05	4.6E+05	4.6E+05	3.2E+05	6.5E+05	4.7E+05	4.6E+05	4.7E+05	7.3E+05	3.2E+05	4.6E+05	4.7E+05	8.5E+05	3.6E+05	4.3E+05	5.0E+05
106	1.6E+05	1.2E+05	1.2E+05	9.4E+04	1.8E+05	1.2E+05	1.2E+05	1.3E+05	2.1E+05	8.1E+04	1.2E+05	1.3E+05	2.4E+05	8.9E+04	1.1E+05	1.3E+05
107	3.0E+05	2.3E+05	2.3E+05	1.4E+05	3.3E+05	2.3E+05	2.1E+05	2.3E+05	3.8E+05	1.7E+05	2.3E+05	2.3E+05	4.4E+05	1.8E+05	2.0E+05	2.4E+05
108	4.4E+05	3.4E+05	3.4E+05	1.9E+05	4.8E+05	3.5E+05	3.2E+05	3.4E+05	5.5E+05	2.9E+05	3.6E+05	3.4E+05	6.3E+05	3.0E+05	3.1E+05	3.3E+05
109	1.5E+05	1.2E+05	1.2E+05	8.0E+04	1.7E+05	1.3E+05	1.1E+05	1.2E+05	1.9E+05	1.0E+05	1.3E+05	1.1E+05	2.2E+05	1.1E+05	1.1E+05	1.0E+05
110	4.0E+05	3.3E+05	3.3E+05	2.3E+05	4.5E+05	3.4E+05	3.3E+05	3.3E+05	5.0E+05	2.2E+05	3.3E+05	3.4E+05	5.8E+05	2.5E+05	3.1E+05	3.6E+05
111	2.7E+06	2.5E+06	2.6E+06	1.7E+06	2.9E+06	2.6E+06	2.5E+06	2.6E+06	3.3E+06	2.2E+06	2.6E+06	2.6E+06	3.9E+06	2.1E+06	2.2E+06	2.6E+06
112	1.1E+06	1.3E+06	1.3E+06	1.1E+06	1.3E+06	1.4E+06	1.4E+06	1.4E+06	1.4E+06	1.2E+06	1.4E+06	1.4E+06	1.7E+06	1.0E+06	1.0E+06	1.2E+06
113	1.8E+06	1.4E+06	1.7E+06	9.7E+05	2.0E+06	1.4E+06	1.4E+06	1.4E+06	2.2E+06	1.2E+06	1.4E+06	1.5E+06	2.6E+06	1.1E+06	1.2E+06	1.4E+06
114	5.4E+05	2.8E+05	4.6E+05	1.4E+05	5.9E+05	2.4E+05	2.3E+05	2.9E+05	6.7E+05	2.2E+05	2.8E+05	2.8E+05	7.8E+05	2.0E+05	2.6E+05	3.0E+05
115	3.0E+05	1.6E+05	2.5E+05	7.6E+04	3.3E+05	1.2E+05	1.2E+05	1.5E+05	3.7E+05	1.2E+05	1.5E+05	1.5E+05	4.3E+05	1.1E+05	1.4E+05	1.7E+05
116	4.0E+05	4.6E+05	4.5E+05	3.5E+05	4.4E+05	4.6E+05	4.6E+05	4.6E+05	5.0E+05	4.2E+05	4.6E+05	4.7E+05	5.8E+05	3.4E+05	3.5E+05	3.9E+05

117	1.5E+06	1.6E+06	1.6E+06	1.2E+06	1.7E+06	1.6E+06	1.5E+06	1.6E+06	1.9E+06	1.3E+06	1.6E+06	1.6E+06	2.2E+06	1.3E+06	1.3E+06	1.5E+06
118	3.7E+05	3.6E+05	3.6E+05	2.5E+05	4.1E+05	3.2E+05	2.8E+05	3.4E+05	4.6E+05	2.9E+05	3.6E+05	3.6E+05	5.4E+05	3.1E+05	3.3E+05	3.2E+05
119	3.6E+04	2.7E+04	3.4E+04	1.6E+04	4.0E+04	2.4E+04	2.0E+04	2.6E+04	4.5E+04	2.1E+04	2.6E+04	2.7E+04	5.2E+04	2.1E+04	2.4E+04	2.6E+04
120	5.3E+04	2.5E+04	3.9E+04	1.0E+04	5.9E+04	2.0E+04	1.8E+04	2.3E+04	6.6E+04	1.7E+04	2.2E+04	2.2E+04	7.7E+04	1.7E+04	2.2E+04	2.5E+04
121	2.6E+05	2.4E+05	2.3E+05	1.0E+05	2.9E+05	2.0E+05	2.3E+05	2.3E+05	3.2E+05	1.3E+05	2.4E+05	2.3E+05	3.7E+05	1.6E+05	1.7E+05	2.3E+05
122	2.7E+05	2.5E+05	2.5E+05	1.5E+05	2.9E+05	2.3E+05	2.5E+05	2.5E+05	3.3E+05	1.6E+05	2.4E+05	2.5E+05	3.9E+05	1.7E+05	1.9E+05	2.3E+05
123	6.8E+05	6.0E+05	6.0E+05	4.5E+05	7.5E+05	6.1E+05	6.1E+05	5.9E+05	8.5E+05	4.7E+05	5.9E+05	6.0E+05	9.8E+05	4.8E+05	5.1E+05	5.6E+05
124	5.5E+05	4.8E+05	4.9E+05	3.0E+05	6.1E+05	5.0E+05	4.7E+05	4.9E+05	6.9E+05	4.2E+05	5.3E+05	5.1E+05	8.0E+05	4.4E+05	4.4E+05	5.2E+05
125	8.3E+05	1.1E+06	1.1E+06	6.8E+05	9.2E+05	1.1E+06	9.9E+05	1.1E+06	1.0E+06	9.3E+05	1.2E+06	1.1E+06	1.2E+06	1.0E+06	1.0E+06	1.1E+06
126	4.1E+05	4.2E+05	4.1E+05	2.9E+05	4.6E+05	3.7E+05	3.2E+05	3.9E+05	5.2E+05	3.1E+05	4.1E+05	4.1E+05	6.0E+05	3.7E+05	3.6E+05	3.7E+05
127	4.5E+05	3.9E+05	3.9E+05	2.8E+05	4.9E+05	3.5E+05	3.0E+05	3.6E+05	5.6E+05	3.1E+05	3.9E+05	3.9E+05	6.5E+05	3.4E+05	3.6E+05	3.5E+05
128	4.9E+05	5.0E+05	5.1E+05	3.4E+05	5.4E+05	4.6E+05	4.4E+05	4.8E+05	6.1E+05	3.8E+05	5.1E+05	5.1E+05	7.1E+05	4.3E+05	4.7E+05	4.6E+05
129	5.4E+04	5.2E+04	5.3E+04	3.5E+04	6.0E+04	4.7E+04	3.9E+04	4.9E+04	6.7E+04	4.2E+04	5.3E+04	5.5E+04	7.8E+04	4.3E+04	4.6E+04	4.7E+04
130	6.4E+04	6.0E+04	6.1E+04	4.0E+04	7.2E+04	5.4E+04	4.4E+04	5.6E+04	8.1E+04	4.8E+04	6.0E+04	6.2E+04	9.4E+04	4.9E+04	5.3E+04	5.4E+04
131	2.3E+05	2.1E+05	2.1E+05	9.5E+04	2.6E+05	1.9E+05	2.1E+05	2.1E+05	2.9E+05	1.3E+05	2.2E+05	2.0E+05	3.4E+05	1.5E+05	1.7E+05	2.3E+05
132	5.6E+05	5.3E+05	5.2E+05	3.3E+05	6.2E+05	4.9E+05	5.2E+05	5.2E+05	7.0E+05	3.4E+05	5.0E+05	5.2E+05	8.1E+05	3.6E+05	4.0E+05	4.8E+05
133	1.2E+06	1.0E+06	1.1E+06	5.4E+05	1.3E+06	1.1E+06	1.1E+06	1.1E+06	1.4E+06	8.3E+05	1.2E+06	1.0E+06	1.7E+06	8.6E+05	1.0E+06	1.1E+06
134	5.3E+05	5.2E+05	5.3E+05	2.8E+05	5.8E+05	5.8E+05	5.3E+05	5.6E+05	6.6E+05	4.7E+05	5.5E+05	5.0E+05	7.6E+05	4.7E+05	4.8E+05	5.2E+05
135	6.5E+05	7.0E+05	7.1E+05	3.0E+05	7.2E+05	7.4E+05	7.3E+05	7.4E+05	8.1E+05	6.3E+05	7.5E+05	7.1E+05	9.4E+05	6.2E+05	6.7E+05	7.4E+05
136	7.6E+05	6.8E+05	6.8E+05	2.0E+05	8.5E+05	6.7E+05	7.0E+05	7.1E+05	9.6E+05	6.0E+05	7.2E+05	7.2E+05	1.1E+06	6.2E+05	6.6E+05	7.3E+05
137	8.0E+05	7.0E+05	6.9E+05	3.4E+05	8.9E+05	7.5E+05	6.9E+05	7.3E+05	1.0E+06	5.4E+05	7.9E+05	7.0E+05	1.2E+06	6.2E+05	7.0E+05	7.5E+05
138	5.1E+05	4.8E+05	4.8E+05	1.7E+05	5.7E+05	4.7E+05	4.5E+05	5.0E+05	6.4E+05	3.6E+05	5.0E+05	4.9E+05	7.4E+05	3.8E+05	4.2E+05	4.7E+05
139	9.8E+05	9.8E+05	9.8E+05	2.4E+05	1.1E+06	9.4E+05	9.5E+05	1.0E+06	1.2E+06	7.7E+05	9.9E+05	1.0E+06	1.4E+06	7.9E+05	8.8E+05	1.0E+06
140	6.7E+05	5.7E+05	5.5E+05	3.3E+05	7.5E+05	5.6E+05	5.5E+05	5.7E+05	8.4E+05	4.4E+05	6.3E+05	5.7E+05	9.8E+05	5.1E+05	5.7E+05	5.4E+05
141	1.5E+06	1.4E+06	1.4E+06	1.1E+06	1.7E+06	1.3E+06	1.5E+06	1.5E+06	1.9E+06	1.0E+06	1.5E+06	1.5E+06	2.2E+06	9.9E+05	1.4E+06	1.3E+06
142	1.5E+05	1.3E+05	1.3E+05	1.1E+05	1.6E+05	7.3E+04	1.2E+05	1.3E+05	1.8E+05	8.7E+04	1.3E+05	1.4E+05	2.1E+05	1.1E+05	1.3E+05	1.3E+05
143	4.7E+04	3.7E+04	3.8E+04	7.8E+03	5.2E+04	3.6E+04	3.6E+04	3.9E+04	5.9E+04	2.9E+04	3.7E+04	3.9E+04	6.8E+04	2.8E+04	3.1E+04	3.5E+04
144	3.8E+04	3.0E+04	3.0E+04	6.3E+03	4.2E+04	2.9E+04	2.9E+04	3.1E+04	4.7E+04	2.4E+04	3.0E+04	3.1E+04	5.5E+04	2.2E+04	2.5E+04	2.8E+04
145	2.3E+04	1.8E+04	1.8E+04	3.8E+03	2.5E+04	1.8E+04	1.8E+04	1.9E+04	2.8E+04	1.4E+04	1.8E+04	1.9E+04	3.3E+04	1.4E+04	1.5E+04	1.7E+04
146	4.9E+04	3.9E+04	4.0E+04	8.2E+03	5.5E+04	3.8E+04	3.8E+04	4.1E+04	6.1E+04	3.1E+04	3.9E+04	4.1E+04	7.1E+04	2.9E+04	3.3E+04	3.6E+04
147	7.4E+04	5.9E+04	6.0E+04	1.2E+04	8.3E+04	5.7E+04	5.7E+04	6.2E+04	9.3E+04	4.6E+04	5.9E+04	6.2E+04	1.1E+05	4.4E+04	5.0E+04	5.5E+04
148	8.8E+04	7.0E+04	7.1E+04	1.5E+04	9.8E+04	6.7E+04	6.7E+04	7.3E+04	1.1E+05	5.5E+04	7.0E+04	7.3E+04	1.3E+05	5.2E+04	5.9E+04	6.5E+04
149	3.6E+04	3.0E+04	2.9E+04	1.5E+04	4.0E+04	2.6E+04	2.9E+04	3.1E+04	4.5E+04	1.6E+04	2.3E+04	2.8E+04	5.3E+04	1.7E+04	1.9E+04	2.9E+04
150	8.2E+04	6.8E+04	6.8E+04	3.1E+04	9.1E+04	5.6E+04	5.2E+04	6.7E+04	1.0E+05	3.3E+04	5.0E+04	6.3E+04	1.2E+05	3.9E+04	4.3E+04	6.9E+04
151	6.0E+04	6.2E+04	6.0E+04	3.0E+04	6.7E+04	5.0E+04	4.6E+04	5.9E+04	7.5E+04	3.1E+04	4.6E+04	5.9E+04	8.7E+04	3.4E+04	3.9E+04	6.1E+04
152	6.7E+04	7.0E+04	6.8E+04	3.4E+04	7.5E+04	5.6E+04	5.2E+04	6.6E+04	8.4E+04	3.5E+04	5.1E+04	6.6E+04	9.8E+04	3.8E+04	4.3E+04	6.9E+04
153	5.3E+04	4.8E+04	4.6E+04	2.3E+04	5.9E+04	3.8E+04	3.5E+04	4.5E+04	6.7E+04	2.4E+04	3.5E+04	4.5E+04	7.8E+04	2.6E+04	3.0E+04	4.7E+04
154	9.1E+04	7.1E+04	6.9E+04	3.5E+04	1.0E+05	5.7E+04	5.3E+04	6.7E+04	1.1E+05	3.5E+04	5.2E+04	6.8E+04	1.3E+05	3.9E+04	4.4E+04	7.0E+04
155	1.1E+05	4.0E+04	4.1E+04	3.6E+04	1.2E+05	4.2E+04	4.2E+04	4.1E+04	1.4E+05	2.6E+04	4.2E+04	2.7E+04	1.6E+05	2.7E+04	4.9E+04	3.3E+04
156	1.5E+05	1.4E+05	1.4E+05	7.7E+04	1.6E+05	1.5E+05	1.3E+05	1.5E+05	1.8E+05	1.3E+05	1.4E+05	1.3E+05	2.1E+05	1.2E+05	1.2E+05	1.3E+05
157	2.9E+05	2.9E+05	2.9E+05	1.5E+05	3.2E+05	3.0E+05	2.8E+05	3.0E+05	3.6E+05	2.6E+05	2.9E+05	2.6E+05	4.2E+05	2.5E+05	2.5E+05	2.5E+05
158	1.8E+05	9.2E+04	1.5E+05	4.3E+04	2.0E+05	6.8E+04	7.0E+04	8.0E+04	2.3E+05	6.4E+04	8.1E+04	8.6E+04	2.7E+05	6.3E+04	8.2E+04	9.9E+04
159	1.5E+05	7.4E+04	1.2E+05	3.2E+04	1.6E+05	5.4E+04	5.4E+04	6.4E+04	1.8E+05	5.1E+04	6.4E+04	6.9E+04	2.1E+05	5.1E+04	6.6E+04	7.9E+04
160	1.1E+05	9.0E+04	9.0E+04	3.9E+04	1.2E+05	8.5E+04	8.5E+04	8.8E+04	1.3E+05	7.0E+04	9.0E+04	9.2E+04	1.5E+05	7.1E+04	7.9E+04	7.9E+04
161	3.7E+05	4.3E+05	4.3E+05	3.2E+05	4.1E+05	4.3E+05	4.3E+05	4.3E+05	4.6E+05	4.0E+05	4.4E+05	4.4E+05	5.3E+05	3.1E+05	3.2E+05	3.6E+05
162	1.3E+05	1.3E+05	1.3E+05	1.0E+05	1.4E+05	1.3E+05	1.3E+05	1.3E+05	1.6E+05	1.2E+05	1.3E+05	1.4E+05	1.8E+05	9.7E+04	9.9E+04	1.1E+05
163	9.8E+04	9.9E+04	9.9E+04	7.5E+04	1.1E+05	1.0E+05	1.0E+05	1.0E+05	1.2E+05	9.3E+04	1.0E+05	1.0E+05	1.4E+05	7.3E+04	7.6E+04	8.5E+04
164	5.1E+04	2.5E+04	2.4E+04	2.1E+04	5.6E+04	2.2E+04	2.2E+04	2.1E+04	6.4E+04	1.5E+04	2.5E+04	1.7E+04	7.4E+04	1.5E+04	2.9E+04	2.3E+04
165	8.8E+04	4.8E+04	4.7E+04	4.0E+04	9.7E+04	4.3E+04	4.3E+04	4.2E+04	1.1E+05	2.9E+04	4.9E+04	3.2E+04	1.3E+05	3.0E+04	5.5E+04	4.4E+04
166	2.9E+04	7.8E+03	7.8E+03	7.3E+03	3.3E+04	8.0E+03	8.6E+03	8.3E+03	3.7E+04	8.6E+03	9.7E+03	8.7E+03	4.3E+04	8.6E+03	1.1E+04	9.5E+03
167	8.3E+03	2.2E+03	2.2E+03	2.0E+03	9.2E+03	2.2E+03	2.4E+03	2.3E+03	1.0E+04	2.4E+03	2.7E+03	2.4E+03	1.2E+04	2.4E+03	3.1E+03	2.6E+03
168	4.1E+03	1.1E+03	1.1E+03	9.9E+02	4.6E+03	1.1E+03	1.2E+03	1.1E+03	5.2E+03	1.2E+03	1.3E+03	1.2E+03	6.0E+03	1.2E+03	1.5E+03	1.3E+03
169	1.3E+04	1.0E+04	1.0E+04	7.7E+03	1.5E+04	1.0E+04	9.6E+03	1.0E+04	1.7E+04	6.7E+03	9.7E+03	1.0E+04	2.0E+04	7.3E+03	8.9E+03	1.1E+04
170	3.1E+04	2.4E+04	2.4E+04	1.8E+04	3.5E+04	2.4E+04	2.3E+04	2.4E+04	3.9E+04	1.6E+04	2.3E+04	2.5E+04	4.5E+04	1.7E+04	2.1E+04	2.5E+04
171	2.7E+04	2.1E+04	2.1E+04	1.6E+04	3.0E+04	2.1E+04	2.0E+04	2.1E+04	3.4E+04	1.4E+04	2.0E+04	2.1E+04	4.0E+04	1.5E+04	1.8E+04	2.2E+04
172	6.4E+04	5.4E+04	5.3E+04	3.3E+04	7.1E+04	4.9E+04	5.1E+04	5.6E+04	8.0E+04	3.3E+04	5.6E+04	5.3E+04	9.3E+04	3.5E+04	4.2E+04	5.5E+04
173	4.5E+04	4.2E+04	3.9E+04	1.9E+04	5.0E+04	3.5E+04	3.9E+04	4.1E+04	5.7E+04	2.3E+04	4.1E+04	4.1E+04	6.6E+04	2.6E+04	3.0E+04	4.1E+04
174	1.1E+04	1.0E+04	1.0E+04	5.8E+03	1.2E+04	1.1E+04	1.0E+04	1.1E+04	1.4E+04	9.5E+03	1.0E+04	9.8E+03	1.6E+04	9.2E+03	8.9E+03	9.5E+03
175	4.0E+03	3.7E+03	3.7E+03	2.1E+03	4.4E+03	4.1E+03	3.6E+03	3.9E+03	5.0E+03	3.5E+03	3.7E+03	3.6E+03	5.8E+03	3.3E+03	3.2E+03	3.4E+03
176	4.0E+02	3.6E+02	3.6E+02	2.0E+02	4.4E+02	3.9E+02	3.5E+02	3.8E+02	5.0E+02	3.3E+02	3.6E+02	3.4E+02	5.8E+02	3.2E+02	3.1E+02	3.3E+02
177	1.8E+04	1.6E+04	1.6E+04	9.2E+03	1.9E+04	1.8E+04	1.6E+04	1.7E+04	2.2E+04	1.5E+04	1.6E+04	1.6E+04	2.6E+04	1.5E+04	1.4E+04	1.5E+04
178	1.5E+05	1.7E+05	1.7E+05	1.2E+05	1.6E+05	1.9E+05	1.8E+05	1.9E+05	1.8E+05	1.9E+05	1.8E+05	2.0E+05	2.1E+05	2.3E+05	2.0E+05	2.0E+05
179	1.0E+04	9.2E+03	9.3E+03	5.3E+03	1.1E+04	1.0E+04	9.1E+03	9.9E+03	1.3E+04	8.6E+03	9.3E+03	8.9E+03	1.5E+04	8.3E+03	8.1E+03	8.6E+03

180	1.2E+05	1.1E+05	1.1E+05	4.7E+04	1.4E+05	1.0E+05	1.0E+05	1.1E+05	1.5E+05	8.4E+04	1.1E+05	1.1E+05	1.8E+05	8.6E+04	9.5E+04	9.5E+04
181	1.3E+05	1.2E+05	1.3E+05	7.5E+04	1.4E+05	1.2E+05	1.1E+05	1.2E+05	1.6E+05	9.2E+04	1.3E+05	1.3E+05	1.9E+05	9.9E+04	1.1E+05	1.1E+05
182	6.2E+04	4.8E+04	4.7E+04	2.3E+04	6.8E+04	4.9E+04	4.4E+04	4.8E+04	7.7E+04	4.0E+04	4.9E+04	4.8E+04	8.9E+04	3.9E+04	4.3E+04	4.9E+04
183	6.8E+03	5.1E+03	5.1E+03	3.8E+03	7.5E+03	5.1E+03	4.8E+03	5.0E+03	8.5E+03	4.4E+03	5.1E+03	5.3E+03	9.8E+03	4.2E+03	4.2E+03	5.0E+03
184	3.9E+04	4.3E+04	4.3E+04	3.7E+04	4.3E+04	4.3E+04	4.2E+04	3.7E+04	4.9E+04	3.1E+04	3.5E+04	3.8E+04	5.7E+04	2.7E+04	3.0E+04	3.5E+04
185	1.7E+04	1.4E+04	1.4E+04	7.0E+03	1.9E+04	1.4E+04	1.3E+04	1.5E+04	2.2E+04	1.1E+04	1.5E+04	1.4E+04	2.5E+04	1.3E+04	1.3E+04	1.4E+04
186	7.3E+03	3.5E+03	3.5E+03	2.8E+03	8.1E+03	3.8E+03	3.7E+03	3.8E+03	9.1E+03	2.8E+03	3.6E+03	3.1E+03	1.1E+04	3.4E+03	4.2E+03	3.5E+03
187	1.2E+05	1.1E+05	1.0E+05	7.4E+04	1.4E+05	7.2E+04	9.3E+04	1.2E+05	1.6E+05	5.5E+04	1.0E+05	1.1E+05	1.8E+05	6.5E+04	7.4E+04	1.1E+05
188	1.2E+05	9.6E+04	9.3E+04	6.7E+04	1.3E+05	6.6E+04	8.4E+04	1.1E+05	1.5E+05	5.0E+04	9.0E+04	1.0E+05	1.7E+05	5.9E+04	6.7E+04	1.0E+05
189	3.7E+03	3.4E+03	3.3E+03	2.4E+03	4.1E+03	2.8E+03	3.3E+03	3.9E+03	4.6E+03	2.1E+03	3.4E+03	3.7E+03	5.4E+03	2.1E+03	2.6E+03	3.6E+03
190	8.9E+03	6.2E+03	6.0E+03	4.1E+03	9.9E+03	4.9E+03	5.2E+03	7.3E+03	1.1E+04	4.1E+03	6.1E+03	5.6E+03	1.3E+04	4.0E+03	5.2E+03	7.1E+03
191	7.0E+04	5.0E+04	4.9E+04	3.3E+04	7.8E+04	3.9E+04	4.2E+04	5.9E+04	8.7E+04	3.3E+04	4.9E+04	4.6E+04	1.0E+05	3.3E+04	4.2E+04	5.8E+04
192	3.9E+04	3.4E+04	3.4E+04	2.9E+04	4.3E+04	3.4E+04	3.3E+04	2.9E+04	4.8E+04	2.4E+04	2.8E+04	3.0E+04	5.6E+04	2.2E+04	2.4E+04	2.8E+04
193	1.4E+05	6.5E+04	1.1E+05	3.1E+04	1.5E+05	4.8E+04	5.0E+04	5.7E+04	1.7E+05	4.5E+04	5.8E+04	6.2E+04	2.0E+05	4.5E+04	5.9E+04	7.1E+04
194	1.1E+04	8.6E+03	8.4E+03	4.2E+03	1.2E+04	8.7E+03	7.8E+03	8.8E+03	1.3E+04	6.6E+03	8.9E+03	8.6E+03	1.5E+04	7.0E+03	7.9E+03	8.2E+03
195	3.1E+04	2.9E+04	2.8E+04	1.4E+04	3.4E+04	2.7E+04	2.6E+04	3.0E+04	3.8E+04	1.8E+04	3.0E+04	2.9E+04	4.5E+04	1.9E+04	2.7E+04	2.9E+04
196	2.9E+05	2.7E+05	2.7E+05	1.9E+05	3.2E+05	2.4E+05	2.0E+05	2.5E+05	3.6E+05	2.0E+05	2.6E+05	2.7E+05	4.2E+05	2.4E+05	2.3E+05	2.4E+05
197	1.4E+05	1.4E+05	1.4E+05	5.2E+04	1.6E+05	1.4E+05	1.3E+05	1.4E+05	1.8E+05	1.2E+05	1.4E+05	1.4E+05	2.1E+05	1.1E+05	1.2E+05	1.3E+05
198	1.5E+04	1.4E+04	1.4E+04	1.2E+04	1.6E+04	1.4E+04	1.4E+04	1.2E+04	1.8E+04	1.0E+04	1.2E+04	1.3E+04	2.1E+04	9.1E+03	1.0E+04	1.2E+04
199	8.3E+04	7.7E+04	7.1E+04	3.6E+04	9.2E+04	6.4E+04	7.2E+04	7.5E+04	1.0E+05	4.3E+04	7.5E+04	7.5E+04	1.2E+05	4.8E+04	5.5E+04	7.5E+04
200	6.6E+02	6.1E+02	6.0E+02	4.4E+02	7.3E+02	5.5E+02	5.8E+02	6.2E+02	8.2E+02	3.8E+02	6.1E+02	6.0E+02	9.6E+02	3.8E+02	4.7E+02	6.0E+02

Table S12. Corridor Index.

Corridor Index	Corridor
1	Seattle, WA -Portland, OR
2	Northwest Sacramento, CA -Portland, OR
3	Southwest Sacramento, CA -Manteca, CA
4	Wheeler Ridge, CA -Manteca, CA
5	Wheeler Ridge, CA -Commerce, CA
6	Irvine, CA -San Diego, CA
7	Southwest Sacramento, CA -Wheeler Ridge, CA
8	Oakland, CA -West Sacramento, CA
9	Oakland, CA -Manteca, CA
10	Pocatello, ID -Butte, MT
11	Pocatello, ID -Salt Lake City, UT
12	South Salt Lake City, UT -Sulphurdale, UT
13	Barstow, CA -Sulphurdale, UT
14	Barstow, CA -San Diego, CA
15	Flagstaff, AZ -Phoenix, AZ
16	Cheyenne, WY -Buffalo, WY
17	Cheyenne, WY -Albuquerque, NM
18	Albuquerque, NM -Denver, CO
19	Las Cruces, NM -Albuquerque, NM
20	Arden Hills, MN -Albert Lea, MN
21	Albert Lea, MN -Ankeny, IA
22	West Des Moines, IA -Northeast Kansas City, MO
23	Northwest Kansas City, MO -Northeast Oklahoma City, OK
24	Southeast Oklahoma City, OK -Northwest Dallas, TX
25	Northeast San Antonio, TX -South Dallas, TX
26	Downtown San Antonio, TX -Corpus Christi, TX
27	Seattle, WA -Butte, MT
28	Butte, MT -Buffalo, WY
29	Buffalo, WY -Albert Lea, MN
30	Portland, OR -Pocatello, ID
31	Northwest Sacramento, CA -Salt Lake City, UT
32	South Salt Lake City, UT -Cheyenne, WY
33	Cheyenne, WY -West Des Moines, IA
34	Sulphurdale, UT -Denver, CO
35	Denver, CO -Northwest Kansas City, MO
36	Barstow, CA -Flagstaff, AZ
37	Flagstaff, AZ -Albuquerque, NM
38	Albuquerque, NM -Southeast Oklahoma City, OK
39	East Oklahoma City, OK -Little Rock, AR
40	Mesquite, TX -Little Rock, AR

41	Reeves County, TX -South Dallas, TX
42	Commerce, CA -Phoenix, AZ
43	Phoenix, AZ -Las Cruces, NM
44	Las Cruces, NM -Reeves County, TX
45	Reeves County, TX -Downtown San Antonio, TX
46	Northeast San Antonio, TX -Houston, TX
47	Houston, TX -Hutchins, TX
48	Arden Hills, MN -Tomah, WI
49	Albert Lea, MN -Tomah, WI
50	Ankeny, IA -La Salle, IL
51	Northeast Kansas City, MO -East St. Louis, MO
52	Northeast Oklahoma City, OK -South St. Louis, MO
53	Little Rock, AR -Northwest Memphis, AR
54	Balch Springs, TX -Southwest Jackson, MS
55	Houston, TX -New Orleans, LA
56	Cherry Valley, IL -La Salle, IL
57	La Salle, IL -Normal, IL
58	Normal, IL -Indian Head Park, IL
59	Bloomington, IL -Troy, IL
60	South St. Louis, MO -Sikeston, MO
61	Sikeston, MO -Northwest Memphis, AR
62	Northeast West Memphis, AR -Southeast Jackson, MS
63	Southwest Jackson, MS -New Orleans, LA
64	Country Club Hills, IL -Champaign, IL
65	Champaign, IL -Jackson Township, IL
66	Jackson Township, IL -Sikeston, MO
67	Gary, IN -Downtown Indianapolis, IN
68	Southeast Indianapolis, IN -Louisville, KY
69	Louisville, KY -North Nashville, TN
70	South Nashville, TN -Downtown Birmingham, AL
71	Downtown Birmingham, AL -Montgomery, AL
72	Montgomery, AL -Mobile, AL
73	Marshall, MI -Northeast Indianapolis, IN
74	Detroit, MI -Perrysburg, OH
75	Perrysburg, OH -Cincinnati, OH
76	Knoxville, TN -Walton, KY
77	Oak Ridge, TN -Chattanooga, TN
78	Chattanooga, TN -Vinings, GA
79	White Springs, FL -Forest Park, GA
80	Naples, FL -White Springs, FL
81	Naples, FL -Fort Lauderdale, FL
82	Southeast Nashville, TN -Wildwood, GA
83	Wildwood, GA -Northeast Birmingham, AL
84	Montgomery, AL -South Fulton, GA
85	Doraville, GA -Statesville, NC

86	Louisville, KY -Walton, KY
87	Cincinnati, OH -Southeast Columbus, OH
88	Southeast Columbus, OH -Brecksville, OH
89	Jackson Township, OH -New Stanton, PA
90	Syracuse, NY -Wapwallopen, PA
91	Wapwallopen, PA -Jonestown, PA
92	Dandridge, TN -Carlisle, PA
93	Jonestown, PA -Bedminster, NJ
94	Albany, NY -Suffern, NY
95	Portland, ME -Salisbury, MA
96	Sturbridge, MA -Rye, NY
97	Rye, NY -Edison, NJ
98	Edison, NJ -Bellmawr, NJ
99	Bellmawr, NJ -Baltimore, MD
100	Springfield, VA -Benson, NC
101	Benson, NC -Florence, SC
102	Jacksonville, FL -Florence, SC
103	Fort Lauderdale, FL -Jacksonville, FL
104	Tomah, WI -Rockford, IL
105	Rockford, IL -Indian Head Park, IL
106	Indian Head Park, IL -Markham, IL
107	Markham, IL -Gary, IN
108	Lake Station, IN -Marshall, MI
109	Marshall, MI -Detroit, MI
110	La Salle, IL -Country Club Hills, IL
111	Lake Station, IN -Perrysburg, OH
112	Perrysburg, OH -Elyria, OH
113	Willoughby Hills, OH -Syracuse, NY
114	Syracuse, NY -Albany, NY
115	Albany, NY -Sturbridge, MA
116	Boston Township, OH -Jackson Township, OH
117	Jackson Township, OH -Wapwallopen, PA
118	Wapwallopen, PA -Parsippany, NJ
119	Suffern, NY -Parsippany, NJ
120	Suffern, NY -Rye, NY
121	Bloomington, IL -Champaign, IL
122	Champaign, IL -Southwest Indianapolis, IN
123	Northeast Indianapolis, IN -Southwest Columbus, OH
124	Southeast Columbus, OH -New Stanton, PA
125	New Stanton, PA -Breezewood, PA
126	Breezewood, PA -Carlisle, PA
127	Carlisle, PA -Bellmawr, NJ
128	Breezewood, PA -Baltimore, MD
129	Parsippany, NJ -Bedminster, NJ
130	Bedminster, NJ -Edison, NJ

131	Troy, IL -Southwest Effingham, IL
132	Northwest Effingham, IL -Southwest Indianapolis, IN
133	Northeast West Memphis, AR -Northwest Nashville, TN
134	Southeast Nashville, TN -Oak Ridge, TN
135	Dandridge, TN -Statesville, NC
136	Statesville, NC -Benson, NC
137	Richland, MS -Downtown Birmingham, AL
138	Northeast Birmingham, AL -Atlanta, GA
139	Panthersville, GA -Florence, SC
140	New Orleans, LA -Mobile, AL
141	Mobile, AL -White Springs, FL
142	White Springs, FL -Jacksonville, FL
143	Vinings, GA -Atlanta, GA
144	Atlanta, GA -South Fulton, GA
145	South Fulton, GA -Forest Park, GA
146	Forest Park, GA -Panthersville, GA
147	Doraville, GA -Panthersville, GA
148	Vinings, GA -Doraville, GA
149	Northeast San Antonio, TX -Downtown San Antonio, TX
150	Northwest Dallas, TX -South Dallas, TX
151	South Dallas, TX -Hutchins, TX
152	Hutchins, TX -Balch Springs, TX
153	Mesquite, TX -Balch Springs, TX
154	Northeast Dallas, TX -Mesquite, TX
155	Commerce, CA -Long Beach, CA
156	Lenoir City, TN -Knoxville, TN
157	Knoxville, TN -Dandridge, TN
158	Salisbury, MA -Hopkinton, MA
159	Hopkinton, MA -Sturbridge, MA
160	College Park, MD -Alexandria, VA
161	Elyria, OH -Brecksville, OH
162	Elyria, OH -Willoughby Hills, OH
163	Willoughby Hills, OH -Brecksville, OH
164	Commerce, CA -Irvine, CA
165	Long Beach, CA -Irvine, CA
166	Northwest Sacramento, CA -Southwest Sacramento, CA
167	West Sacramento, CA -Northwest Sacramento, CA
168	West Sacramento, CA -Southwest Sacramento, CA
169	Markham, IL -Country Club Hills, IL
170	Country Club Hills, IL -Homewood, IL
171	Markham, IL -Hazel Crest, IL
172	East St. Louis, MO -Troy, IL
173	Northwest Effingham, IL -Southwest Effingham, IL
174	North Nashville, TN -Northwest Nashville, TN
175	Northwest Nashville, TN -South Nashville, TN

176	South Nashville, TN -Southeast Nashville, TN
177	North Nashville, TN -Southeast Nashville, TN
178	Cincinnati, OH -Walton, KY
179	Southeast Nashville, TN -Southeast Nashville, TN (I-40)
180	Beltsville, MD -Springfield, VA
181	Baltimore, MD -Beltsville, MD
182	Gary, IN -Lake Station, IN
183	Southwest Columbus, OH -Southeast Columbus, OH
184	Southwest Indianapolis, IN -South Indianapolis, IN
185	Downtown Birmingham, AL -Northeast Birmingham, AL
186	Salt Lake City, UT -South Salt Lake City, UT
187	Ankeny, IA -West Des Moines, IA
188	Ankeny, IA -West Des Moines, IA
189	Northwest Kansas City, MO -Northeast Kansas City, MO
190	Southeast Oklahoma City, OK -East Oklahoma City, OK
191	Northeast Oklahoma City, OK -East Oklahoma City, OK
192	Downtown Indianapolis, IN -Northeast Indianapolis, IN
193	Salisbury, MA -Hopkinton, MA
194	Southwest Jackson, MS -Southeast Jackson, MS
195	Northwest Memphis, AR -Northeast West Memphis, AR
196	Jonestown, PA -Carlisle, PA
197	Wildwood, GA -Chattanooga, TN
198	Downtown Indianapolis, IN -Southeast Indianapolis, IN
199	Normal, IL -Bloomington, IL
200	South St. Louis, MO -East St. Louis, MO

SI References

(1) Gagnon, P.; Frazier, W.; Cole, W.; Hale, E. *Cambium Documentation: Version 2021*; NREL/TP-6A40-81611; National Renewable Energy Laboratory: Golden, CO, 2021.
(2) US EPA, O. *Emissions & Generation Resource Integrated Database (eGRID)*. https://www.epa.gov/egrid (accessed 2023-02-22).
(3) *Freight Analysis Framework - FHWA Freight Management and Operations*. https://ops.fhwa.dot.gov/freight/freight_analysis/faf/ (accessed 2023-02-22).
(4) Tong, F.; Azevedo, I.; Jaramillo, P. Economic Viability of a Natural Gas Refueling Infrastructure for Long-Haul Trucks. *Journal of Infrastructure Systems* **2019**, *25* (1), 04018039. https://doi.org/10.1061/(ASCE)IS.1943-555X.0000460.
(5) Tong, F.; Jenn, A.; Wolfson, D.; Scown, C. D.; Auffhammer, M. Health and Climate Impacts from Long-Haul Truck Electrification. *Environ. Sci. Technol.* **2021**, *55* (13), 8514–8523. https://doi.org/10.1021/acs.est.1c01273.
(6) Tong, F.; Wolfson, D.; Jenn, A.; Scown, C. D.; Auffhammer, M. Energy Consumption and Charging Load Profiles from Long-Haul Truck Electrification in the United States. *Environ. Res.: Infrastruct. Sustain.* **2021**, *1* (2), 025007. https://doi.org/10.1088/2634-4505/ac186a.
(7) Jenn, A.; Clark-Sutton, K.; Gallaher, M.; Petrusa, J. Environmental Impacts of Extreme Fast Charging. *Environ. Res. Lett.* **2020**, *15* (9), 094060. https://doi.org/10.1088/1748-9326/ab9870.
(8) Stocker, T. F.; Qin, D.; Plattner, G.; Tignor, M. M. B.; Allen, S.; Boschung, J.; Nauels, A.; Xia, Y.; Bex, V.; Midgley, P. M. Climate Change 2013: The Physical Science Basis. Working Group I Contribution to the Fifth Assessment Report of the Intergovernmental Panel on Climate Change, 2014.
(9) Preble, C. V.; Harley, R. A.; Kirchstetter, T. W. Control Technology-Driven Changes to In-Use Heavy-Duty Diesel Truck Emissions of Nitrogenous Species and Related Environmental Impacts. *Environ. Sci. Technol.* **2019**, *53* (24), 14568–14576. https://doi.org/10.1021/acs.est.9b04763.
(10) Argonne National Laboratory (ANL). The GREET (Greenhouse Gases, Regulated Emissions, and Energy Use in Transportation) Model. https://greet.es.anl.gov/.
(11) Tong, F.; Jaramillo, P.; Azevedo, I. M. L. Comparison of Life Cycle Greenhouse Gases from Natural Gas Pathways for Medium and Heavy-Duty Vehicles. *Environ. Sci. Technol.* **2015**, *49* (12), 7123–7133. https://doi.org/10.1021/es5052759.
(12) Guttenberg, M.; Sripad, S.; Viswanathan, V. Evaluating the Potential of Platooning in Lowering the Required Performance Metrics of Li-Ion Batteries to Enable Practical Electric Semi-Trucks. *ACS Energy Lett.* **2017**, *2* (11), 2642–2646. https://doi.org/10.1021/acsenergylett.7b01022.
(13) Tessum, C. W.; Hill, J. D.; Marshall, J. D. Life Cycle Air Quality Impacts of Conventional and Alternative Light-Duty Transportation in the United States. *Proceedings of the National Academy of Sciences* **2014**, *111* (52), 18490–18495. https://doi.org/10.1073/pnas.1406853111.
(14) Interagency Working Group on Social Cost of Greenhouse Gases. *TechnicalSupportDocument_SocialCostofCarbonMethaneNitrousOxide.pdf*. WhiteHouse.gov. https://www.whitehouse.gov/wp-

content/uploads/2021/02/TechnicalSupportDocument_SocialCostofCarbonMethaneNitrousOxide.pdf (accessed 2023-05-15).
(15) Tong, F.; Azevedo, I. M. L. What Are the Best Combinations of Fuel-Vehicle Technologies to Mitigate Climate Change and Air Pollution Effects across the United States? *Environ. Res. Lett.* **2020**, *15* (7), 074046. https://doi.org/10.1088/1748-9326/ab8a85.
(16) Krewski, D.; Jerrett, M.; Burnett, R. T.; Ma, R.; Hughes, E.; Shi, Y.; Turner, M. C.; Pope III, C. A.; Thurston, G.; Calle, E. E.; Thun, M. J. Extended Follow-Up and Spatial Analysis of the American Cancer Society Study Linking Particulate Air Pollution and Mortality, 2009.
(17) Tessum, C. W.; Apte, J. S.; Goodkind, A. L.; Muller, N. Z.; Mullins, K. A.; Paolella, D. A.; Polasky, S.; Springer, N. P.; Thakrar, S. K.; Marshall, J. D.; Hill, J. D. Inequity in Consumption of Goods and Services Adds to Racial–Ethnic Disparities in Air Pollution Exposure. *Proceedings of the National Academy of Sciences* **2019**, *116* (13), 6001–6006. https://doi.org/10.1073/pnas.1818859116.
(18) Gagnon, P.; Cowiestoll, B.; Schwarz, M. *Cambium 2022 Scenario Descriptions and Documentation*; NREL/TP-6A40-84916, 1915250, MainId:85689; 2023; p NREL/TP-6A40-84916, 1915250, MainId:85689. https://doi.org/10.2172/1915250.
(19) Gagnon, P.; Brown, M.; Steinberg, D.; Brown, P.; Awara, S.; Carag, V.; Cohen, S.; Cole, W.; Ho, J.; Inskeep, S.; Lee, N.; Mai, T.; Mowers, M.; Murphy, C.; Sergi, B. 2022 Standard Scenarios Report: A U.S. Electricity Sector Outlook. *Renewable Energy* **2023**.
(20) *Scenario Viewer*. https://scenarioviewer.nrel.gov/ (accessed 2023-03-21).
